# Supplementary material for: Ultrastructural analysis of synapses after induction of spike-timing-dependent plasticity
Source: Cell Rep Methods. 2025 Aug 25;5(9):101142. doi: 10.1016/j.crmeth.2025.101142 (PMC12539246; doi:10.1016/j.crmeth.2025.101142)
Supplement: Document S1. Figures S1–S4 [file mmc1.pdf]

Cell Reports Methods, Volume *5*

Supplemental information

Ultrastructural analysis

# of synapses after induction

# of spike-timing-dependent plasticity

Rui Wang, Michaela Schweizer, Margarita Anisimova, Christine E. Gee, and Thomas G. Oertner

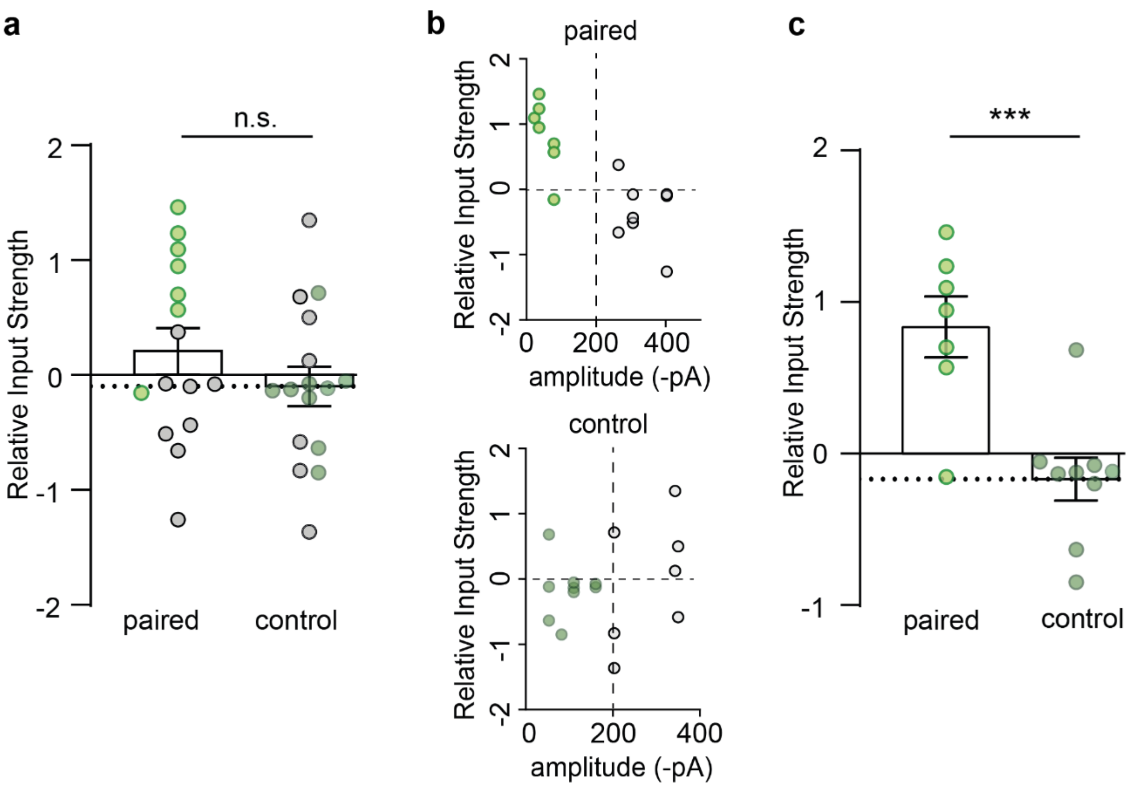

Figure S1. Activating a large number of presynaptic neurons prevents selective potentiation of paired synapses, related to Figure 3.

(**a**) Full dataset. Each dot indicates the EPSC amplitude of a CheRiff-expressing CA1 pyramidal cell relative to 2-3 non-transfected neighbors after causal pairing or without optical stimulation (control). No difference in input strength between the paired and the unpaired group relative to non-transfected neighbors. Green dots: average EPSC amplitude < 200 pA; black dots: average EPSC amplitude > 200 pA. n = 15, 16. Unpaired t-test, ns: not significant.
(**b**) Replot of data in (a) to show the relationship of input strength versus the averaged NT amplitude in paired (upper) and unpaired (lower) groups.
(**c**) All experiments with EPSC amplitude < 200 pA, indicating few ChrimsonR-expressing CA3 neurons. Significant input strengthening relative to non-transfected neighbors in the paired group only. n = 7, 9 neurons. Unpaired t-test, ***p < 0.001. Data plotted as mean ± SEM.

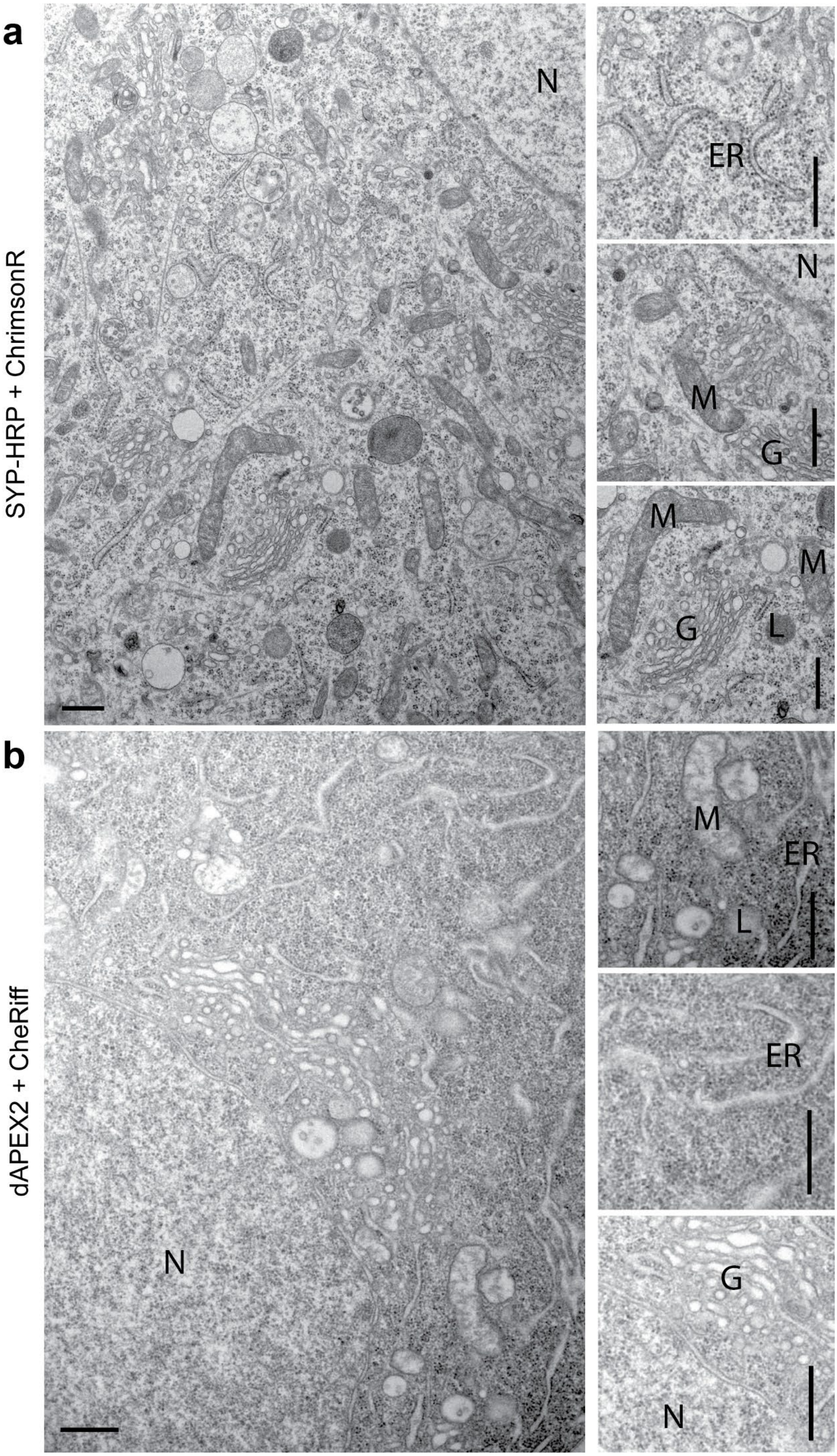
a
SYP-HRP + ChrimsonR
N
ER
N
M
G
M
M
G
L
b
dAPEX2 + CheRiff
N
M
ER
L
ER
G
N

Figure S2. Somatic ultrastructure of double-transduced neurons, related to Figure 4.

(**a**) CA3 pyramidal cell soma expressing SYP-HRP and ChrimsonR. Cytoplasm at the cell body including part of the nucleus (N). Due to synaptic vesicle targeting of HRP, no cytoplasmic DAB staining is visible in this neuron (see Fig. 4). Organelle morphology is well preserved (G: Golgi apparatus, M: mitochondria, L: lysosomes, ER: endoplasmic reticulum, N: nucleus). Scale bars: 500 nm.

(**b**) CA1 pyramidal cell soma expressing dAPEX2 and CheRiff. Membrane contrast is relatively low due to dAPEX2 condensation in the cytoplasm (dark granules). Organelle morphology is well preserved (G: Golgi apparatus, M: mitochondria, L: lysosomes, ER: endoplasmic reticulum, N: nucleus). Scale bars: 500 nm.

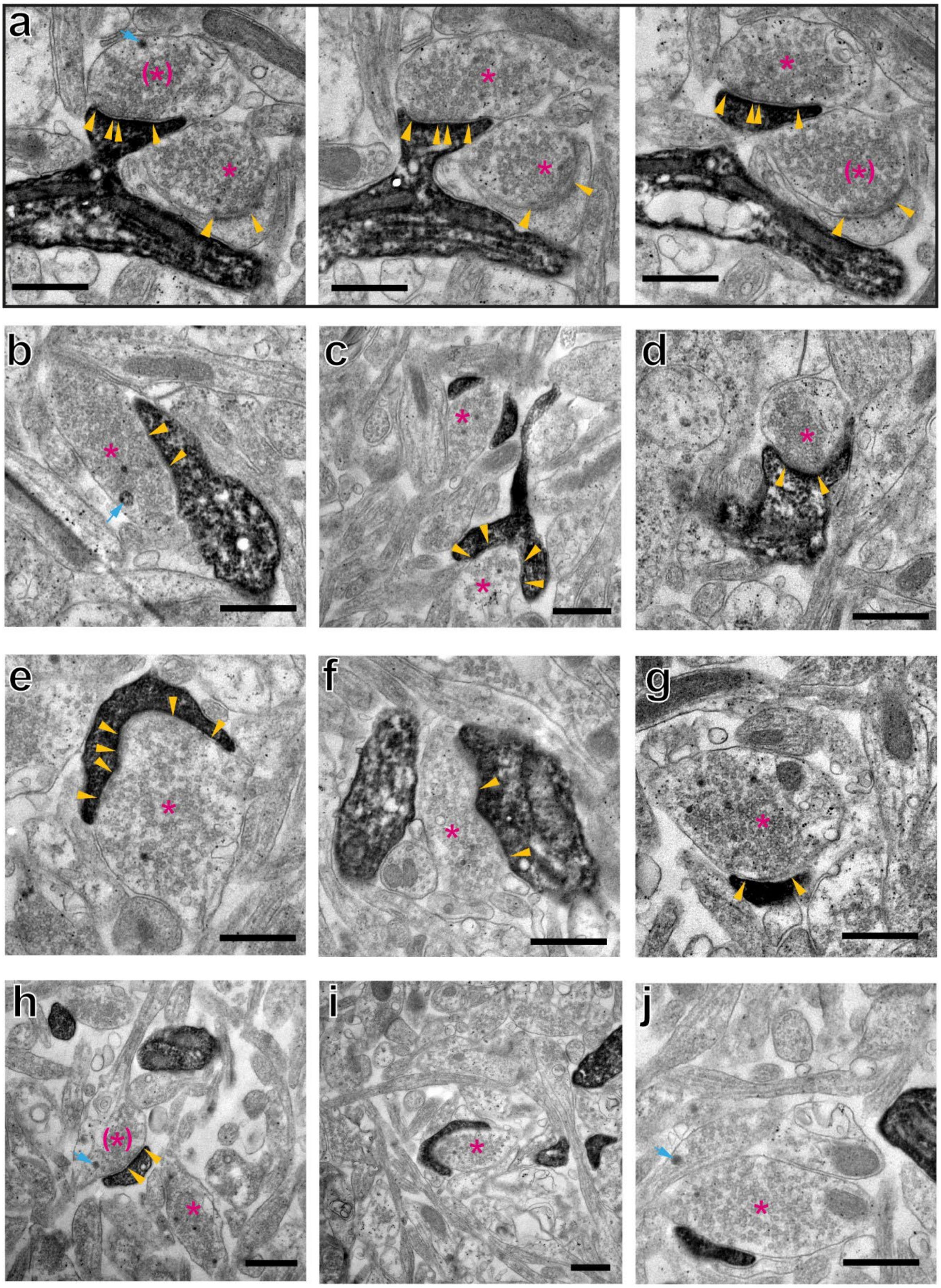

Figure S3. Double-labeled contacts after causal stimulation, related to Figure 4.

(**a**) Two spine synapses identified in 3 consecutive sections. Yellow arrowheads mark edge of PSDs. Magenta asterisks: Stimulated presynaptic terminal containing one or more labeled vesicles. Magenta asterisks in brackets: Stimulated terminal identified by labeled vesicles in other sections. Cyan arrow: dense core vesicle, not indicative of labeling. (**b**) – (**h**) Further examples of double-labeled synapses, identified in single sections. (**i**), (**j**) Putative synapses, PSD not identified. All scale bars: 250 nm.

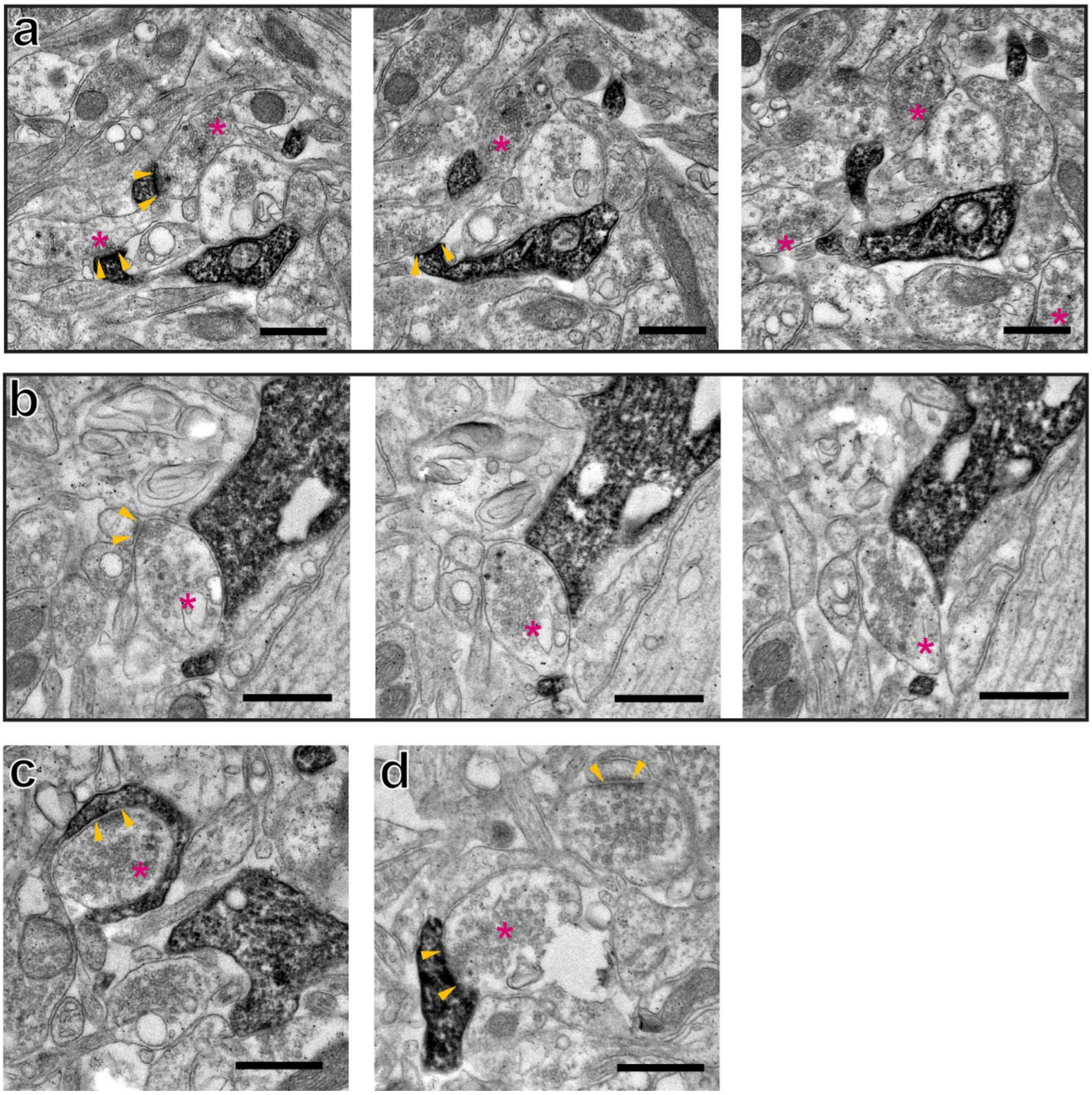

Figure S4. Double-labeled contacts after anti-causal stimulation, related to Figure 4.

(**a**) Two double-labeled spine synapses identified in 3 consecutive sections. Magenta asterisks: Stimulated presynaptic terminal containing one or more labeled vesicles. Yellow arrowheads denote PSD. (**b**) Pre-only synapse (Yellow arrowheads) with contact to a labeled postsynaptic dendrite, identified in 3 consecutive sections. (**c**),(**d**) Further examples of double-labeled synapses. All scale bars: 250 nm.
