# Supplementary material for: Ultrastructural analysis of synapses after induction of spike-timing-dependent plasticity
Source: Cell Rep Methods. 2025 Aug 25;5(9):101142. doi: 10.1016/j.crmeth.2025.101142 (PMC12539246; doi:10.1016/j.crmeth.2025.101142)
Supplement: Document S2. Article plus supplemental information [file mmc2.pdf]

Cell Reports
Methods

Report

Ultrastructural analysis of synapses after induction of spike-timing-dependent plasticity

Graphical abstract

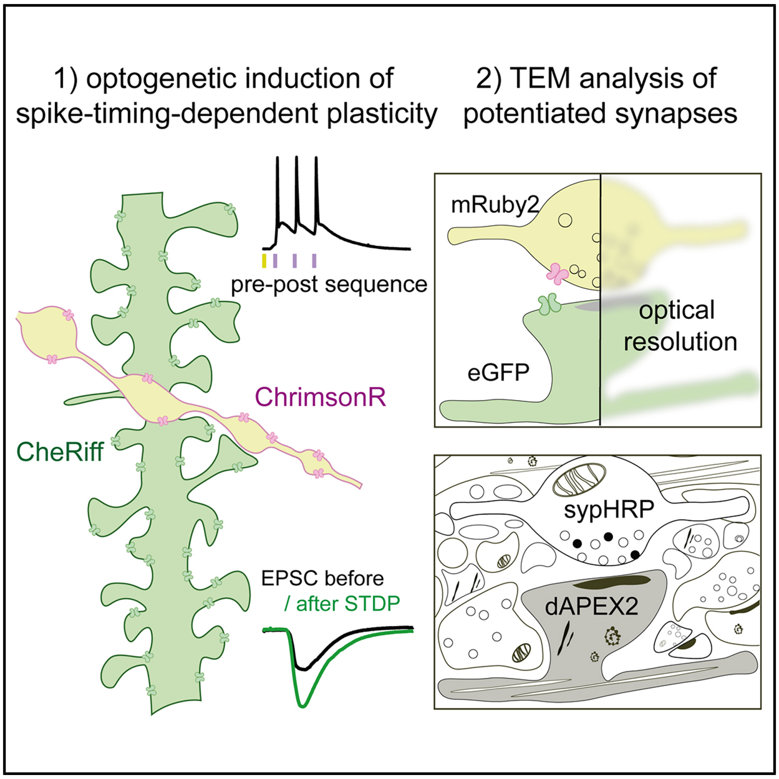

Authors

Rui Wang, Michaela Schweizer, Margarita Anisimova, Christine E. Gee, Thomas G. Oertner

Correspondence

thomas.oertner@zmnh.uni-hamburg.de

In brief

Optogenetic induction of spike-timing-dependent plasticity leads to the strengthening of a few hippocampal synapses among thousands of synapses that received no paired stimulation. Wang et al. combine channelrhodopsins with electron-dense labels to find the potentiated synapses with electron microscopy and show that potentiated spines grow and curve around their presynaptic boutons.

Highlights

- We optogenetically induce spike-timing-dependent plasticity
- We combine channelrhodopsin with electron-dense labels for electron microscopy
- We perform ultrastructural analysis of optogenetically manipulated neurons
- We show that paired stimulation drives expansion of axon-spine interface

Wang et al., 2025, Cell Reports Methods 5, 101142
September 15, 2025 © 2025 The Author(s). Published by Elsevier Inc.
https://doi.org/10.1016/j.crmeth.2025.101142

CellPress

Cell Reports Methods

CellPress
OPEN ACCESS

Report

Ultrastructural analysis of synapses after induction of spike-timing-dependent plasticity

Rui Wang,[1] Michaela Schweizer,[2] Margarita Anisimova,[1] Christine E. Gee,[1] and Thomas G. Oertner[1,3,*]
[1]Institute for Synaptic Neuroscience, ZMNH, University Medical Center Hamburg-Eppendorf, 20251 Hamburg, Germany
[2]Core Facility Morphology & Electron Microscopy, ZMNH, University Medical Center Hamburg-Eppendorf, 20251 Hamburg, Germany
[3]Lead contact
*Correspondence: thomas.oertner@zmnh.uni-hamburg.de
https://doi.org/10.1016/j.crmeth.2025.101142

MOTIVATION Spike-timing-dependent plasticity (STDP) induces changes in the strength of synaptic connections between neurons that have been spiking together. Whether this results in long-lasting changes in the ultrastructure of individual synapses or whether other changes are responsible for synaptic memory has not been investigated. This gap in our understanding is largely due to technical difficulties. First, it has only recently become possible to induce STDP without patching the postsynaptic neuron. Second, it is not trivial to individually label the pre- and postsynaptic neurons that have undergone STDP. Here, we present a workflow that allows all-optical induction of STDP and subsequent processing of the tissue to find the relevant pre- and postsynaptic structures using transmission electron microscopy.

SUMMARY

Repeated sequential activation of connected neurons causes lasting changes in synaptic strength, a process known as spike-timing-dependent plasticity (STDP). Recently, sequential spike patterns have been induced without electrodes, using two spectrally separated channelrhodopsins. However, due to the difficulty of labeling and localizing the few connecting synapses between the stimulated pre- and postsynaptic neurons (~1–5 per neuron pair), ultrastructural analysis after STDP has not been reported. Here, we optogenetically induce STDP at CA3-CA1 hippocampal synapses and identify stimulated boutons and spines in CA1 using transmission electron microscopy (TEM). Presynaptic CA3 neurons express vesicle-targeted horseradish peroxidase, cre recombinase, and cre-dependent ChrimsonR, a red light-activatable channelrhodopsin. Postsynaptic neurons express violet light-activatable CheRiff and dAPEX2, an enhanced ascorbate peroxidase. In TEM, presynaptic boutons and postsynaptic spines are readily identifiable with well-preserved ultrastructural features. Our labeling strategy allows ultrastructural analysis of optogenetically manipulated neurons and their synapses.

INTRODUCTION

Exactly what changes in the brain when a new memory is formed is not fully understood. The memory trace, or "engram," may consist of minute structural changes at the synaptic connections between neurons that have repeatedly fired together. Spike-timing-dependent plasticity (STDP), induced by precisely ordered firing of neurons, causes timing-dependent long-term potentiation (tLTP) or timing-dependent long-term depression (tLTD) of cortical synapses.[1–3] Recently, we have shown that the functional changes induced by optogenetic STDP (oSTDP) in the hippocampus can be very long-lasting.[4] Assessing the strength of synaptic connections days after the induction of plasticity became possible by controlling the firing of pre- and postsynaptic action potentials through light-gated channels (channelrhodopsins) instead of invasive recordings with glass electrodes.

It is unknown whether the relative increase in synaptic strength induced by STDP is maintained by a physical change in synapse size. Multiple synapses formed by boutons from one axon onto spines of a specific postsynaptic neuron have remarkably similar ultrastructures, suggesting that synaptic structure is tightly controlled by the activity history in the presynaptic and postsynaptic neurons.[5–7] However, the actual activity history and strength of the connection between the pre- and postsynaptic neurons were unknown in these studies. Rather than increasing

This is an open access article under the CC BY license (http://creativecommons.org/licenses/by/4.0/).

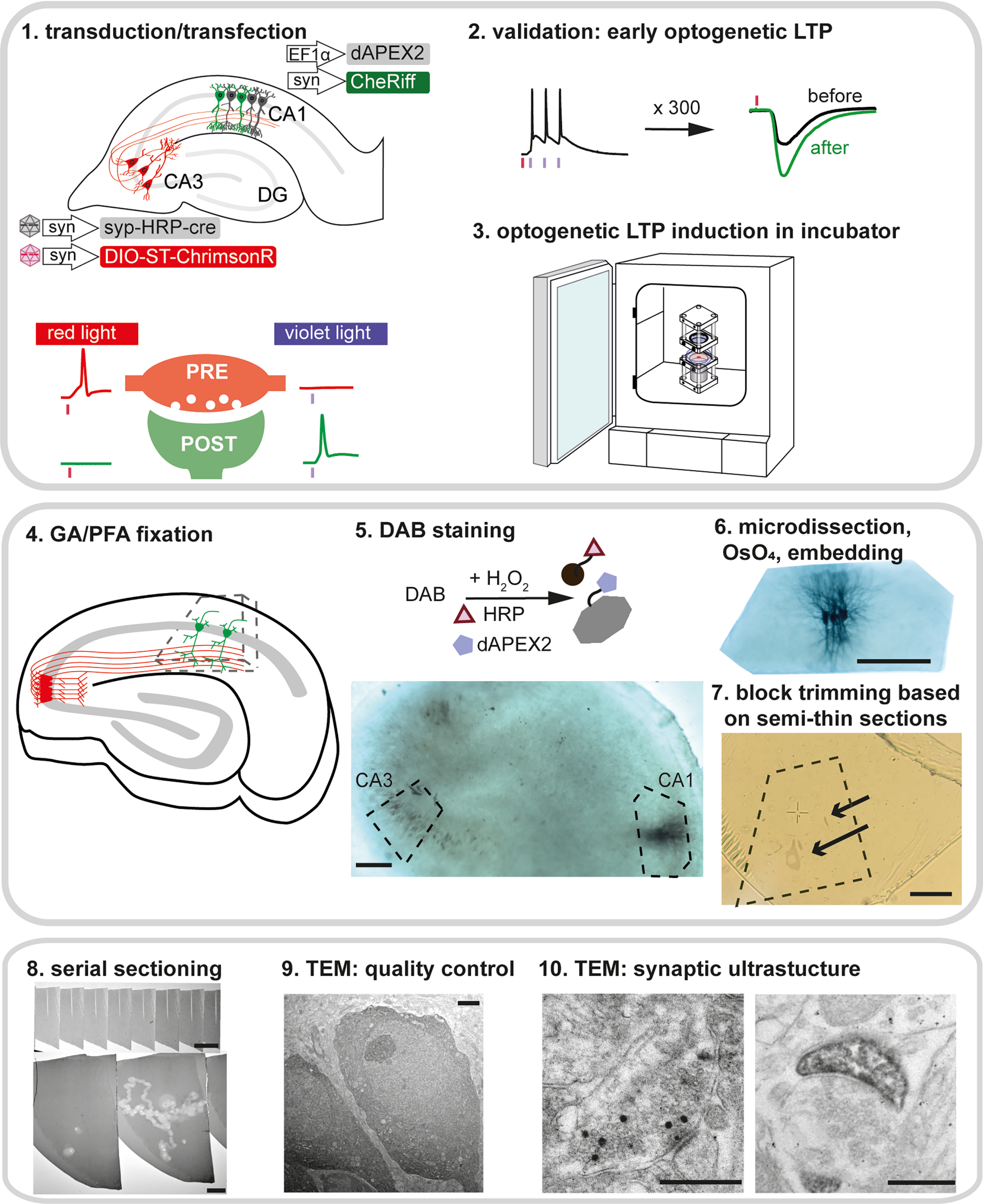

Figure 1. Workflow of STDP induction and sample preparation to study synaptic ultrastructure

Step 1: AAV-assisted transduction of presynaptic neurons and single-cell electroporation to transfect postsynaptic neurons. Red light (635 nm) only induces action potentials in presynaptic neurons expressing ChrimsonR, and violet light (405 nm) only induces action potentials in postsynaptic neurons expressing CheRiff.

Steps 2 and 3: optogenetic induction of STDP. Induction of all-optical tLTP by 300 repetitions of single presynaptic (red) and three postsynaptic (violet) action potentials. Validation of all-optical tLTP at early and late time points.

Step 4: fixation at the desired time point after induction of tLTP.

Step 5: metal-enhanced DAB staining to reveal HRP in presynaptic vesicles and dAPEX2 in the postsynaptic neurons.

Step 6: micro-dissection of the stained CA3 and CA1 regions, osmification, and embedding.

Step 7: further trimming of the block based on DAB-stained somata and processes in semi-thin sections (arrows).

(legend continued on next page)

CellPress
OPEN ACCESS

the size of existing synapses, newly formed (additional) synaptic connections between neurons would also increase the functional strength of the connection. The formation of new spine synapses can be induced by localized glutamate release onto a dendrite.[8,9]

Since the size of a typical CNS synapse is close to the diffraction limit of light, subcellular synaptic structures cannot be visualized by conventional light microscopy. Stimulated emission depletion (STED) microscopy is a promising approach to imaging dendritic spines and associated presynaptic vesicle clusters.[10] However, fluorescence-based methods alone visualize only labeled entities and provide no information about surrounding unlabeled structures or cells in the tissue unless they are combined with electron microscopy (EM).[11] After osmium tetroxide staining, unlabeled membranes become visible in EM, providing precise information about the volume of spines and presynaptic boutons, the size of postsynaptic densities and active zones, and the presence of organelles such as vesicles, mitochondria, and endoplasmic reticulum (ER). Several studies have analyzed synaptic ultrastructures after high-frequency electrical stimulation.[12–15] A limitation of blind electrical stimulation is that just a subset of axons is activated at the desired frequency, leading to a mix of potentiated, partially stimulated, and unstimulated synapses in the analyzed samples.

Correlative light-electron microscopy (CLEM) allows analyzing the ultrastructure of identified synapses that were directly stimulated, e.g., by glutamate uncaging in Mg^{2+}-free artificial cerebrospinal fluid (ACSF).[16] Although there is no activation of the presynaptic terminal in these experiments, the resulting rapid changes in postsynaptic ultrastructure are impressive. Optogenetic stimulation offers the exciting possibility to drive spiking in identified neurons non-invasively under physiological conditions. It has recently been combined with membrane-targeted peroxidase expression to identify light-activated axons by post-embedding immunogold labeling.[17] Our goal was to extend this promising approach to establish a labeling protocol that would allow the detection of STDP-stimulated ("paired") synapses and the investigation of activity-related ultrastructural changes. Paired synapses could then be compared to three types of "control" synapses: (1) nearby synapses between unlabeled/unpaired neurons, (2) synapses from the same presynaptic neuron onto unstimulated postsynaptic neurons, and (3) other synapses onto the same postsynaptic dendrite. Control synapses within the same tissue block are ideal to exclude small variations in fixation or staining that could affect the quantification of synaptic ultrastructure. We present a methodological workflow starting with viral transduction of neurons, induction of oSTDP, and processing of fixed tissue for transmission electron microscopy (TEM) to study ultrastructural changes at synapses associated with tLTP.

We present a combination of opsins, EM-compatible labels, and staining procedures that allows unambiguous identification of presynaptic boutons of ChrimsonR-expressing CA3 neurons and postsynaptic spines and dendrites of CheRiff-expressing CA1 neurons. Since both EM labels are peroxidases that are detected by 3,3′-diaminobenzidine (DAB) staining, our strategy is also compatible with serial block-face imaging, automated 3D reconstruction, and analysis,[18–21] which should greatly improve the throughput and success rate of finding potentiated synapses. Since both labels are genetically expressed, they can be precisely targeted to the cell types of interest and are broadly applicable to studies of other synapses of interest. Postsynaptic dAPEX2 could also be expressed in other cell types, such as astrocytes or microglia, if the goal is to study their proximity to specific synapses. There is no reliance on antibody-mediated labeling, so there are no concerns about tissue penetration, antigen availability, selectivity, or specificity. In addition, fluorescence does not need to be retained during fixation steps, so these can be strong and optimized for the easy identification of membranes and organelles. We expect this method to be particularly useful in answering open questions about the fate of synapses and their ultrastructural changes after STDP.

RESULTS

The ultrastructure of potentiated synapses

Our goal was to induce STDP non-invasively and subsequently examine the ultrastructure of the presynaptic boutons and postsynaptic spines connecting the co-stimulated neurons (Figure 1). The most critical step in the development of this workflow was the identification of a pair of peroxidase-based genetic tags that, after processing and TEM, would allow the unambiguous identification of the structures originating from presynaptic and postsynaptic neurons. We focused on Schaffer collateral synapses formed by axons of CA3 pyramidal cells onto spines of CA1 pyramidal cells in rat organotypic hippocampal slice cultures. Since the slice cultures are ∼300 μm thick, we wanted to avoid antibody-based labeling, which often fails to reveal structures more than ∼50 μm below the surface.

Step 1: Viral transduction of CA3 neurons and single-cell electroporation to transfect CA1 neurons

To independently spike and label presynaptic CA3 neurons, we used two recombinant adeno-associated viral vectors (rAAVs). One rAAV encoded the red light-activatable channelrhodopsin ChrimsonR, the second a synaptic vesicle-targeted horseradish peroxidase (synaptophysin-HRP [syp-HRP]). Initial attempts to package a single plasmid encoding ChrimsonR-tdTomato and syp-HRP into one rAAV were unsuccessful. When we mixed two rAAVs with capsid serotype rh10, the percentage of neurons expressing both proteins was disappointingly low (∼40%). We solved this problem of poor co-expression with the Cre/*LoxP* system.[22] We packaged a cre-dependent version of ChrimsonR in one rAAV and a bicistronic vector for co-expression of syp-HRP and cre (syp-HRP-IRES-cre) in a second one, both with capsid serotype AAV9. We observed that 1 week after local

Step 8: ultrathin serial sectioning for TEM.
Step 9: verify structural preservation/sample quality at the soma area of labeled CA3 and CA1 neurons.
Step 10: locate and image structures of interest.
Scale bars: steps 5 and 6: 200 μm; 7: 20 μm; 8: 60 μm and 20 μm; 9: 2 μm; and 10: 500 nm.

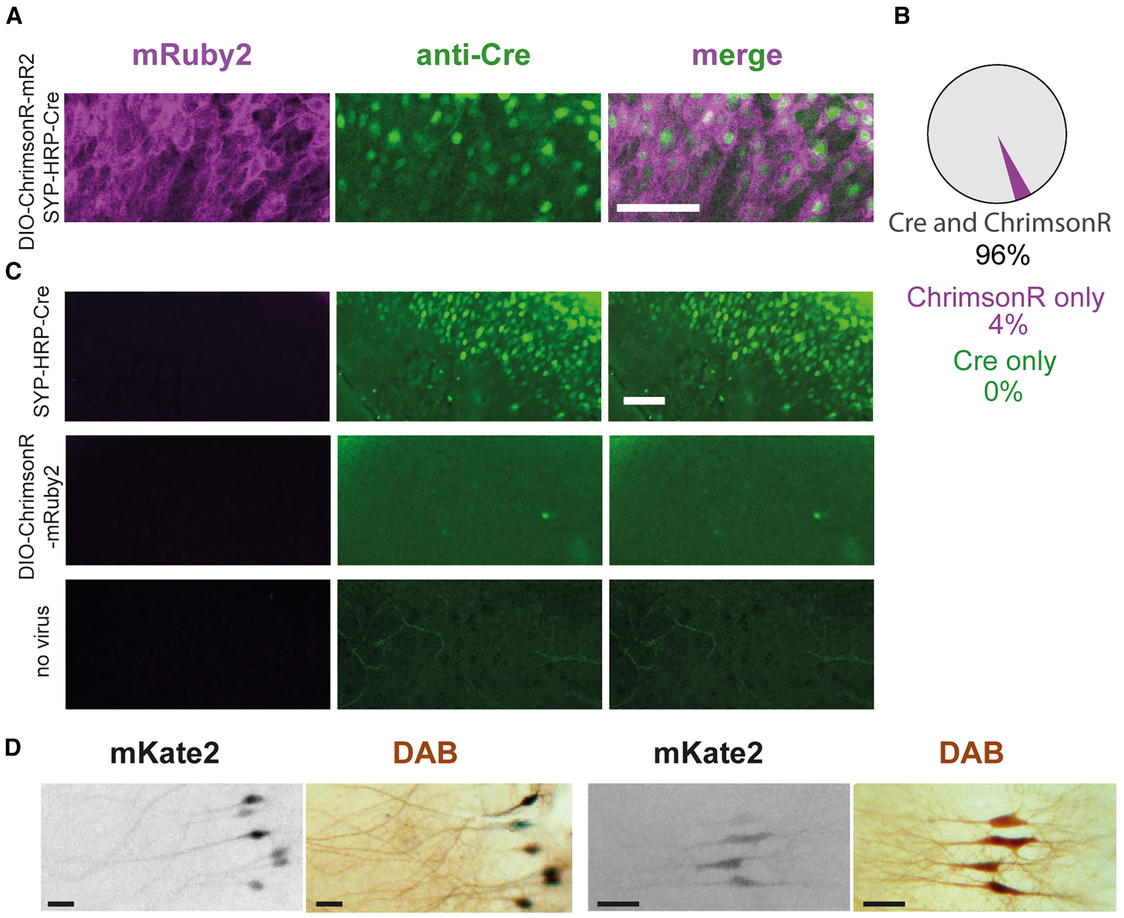

Figure 2. Assessment of electron microscopy label and channelrhodopsin co-expression

(A) CA3: a mixture of rAAV$^{2/9}$-syn-DIO-ChrimsonR-mRuby2-ST and rAAV$^{2/9}$-syn-SYP-HRP-IRES-Cre was co-injected into CA3. Slices were fixed and immunostained against mRuby2 (magenta) and cre (green). Scale bar: 100 μm.

(B) Quantification of co-expression rate of ChrimsonR-mRuby2 and syp-HRP-IRES-Cre. n = 202 cells (4 slice cultures).

(C) Application of the individual viruses or no virus. Scale bar: 100 μm.

(D) Two examples of co-expression in CA1 pyramidal neurons electroporated with plasmids encoding dAPEX2, mKate2, and CheRiff-eGFP: mKate2 fluorescence (contrast-inverted grayscale) several days before fixation and corresponding DAB staining after fixation (brown). Scale bars: 40 μm (left images) and 70 μm (right images).

injection of the mixed rAAVs, 96% of transduced CA3 neurons expressed both ChrimsonR and syp-HRP-IRES-cre (Figures 2A and 2B). The remainder (4%) appeared to express only ChrimsonR, indicating that cre expression was below the antibody detection limit. This interpretation is supported by the observation that no neurons expressed ChrimsonR when the rAAV was applied alone (Figure 2C). Somewhat surprisingly, we detected no neurons expressing only syp-HRP-IRES-cre when the two rAAVs were applied together. Taking these results together, we expect almost no cases where light-activated CA3 neurons do not express the EM label syp-HRP, and vice versa. A small volume of the virus mix was pressure injected into area CA3 of hippocampal cultures using a picospritzer (1.8 bar, 50 ms pulse). As we have previously shown,[4] it is very important that the number of transduced CA3 neurons is in the range of 30–40 to achieve successful and specific induction of oSTDP. Simultaneous activation of a larger number of CA3 neurons causes firing of secondary (non-transfected [NT]) neurons and, consequently, synaptic plasticity at unlabeled synapses. For reliable oSTDP, light-induced excitatory postsynaptic currents (EPSCs) in the postsynaptic neuron should be well below 200 pA (Figure S1).

To independently spike and label a few postsynaptic CA1 neurons, we co-expressed the violet light-activatable channelrhodopsin CheRiff with dAPEX2,[21] an enhanced version of soluble APEX2,[23] by single-cell electroporation of a 1:20 plasmid mixture.[24] A high concentration of dAPEX2 was critical to later trim the resin block close to the dark dendritic branches of electroporated cells. For electrophysiology experiments, a small amount of mKate2 plasmid was added to allow us to visualize the transfected CA1 neurons without activating CheRiff. In our patch-clamp experiments, we never encountered mKate2-expressing neurons without photocurrents, suggesting highly reliable co-expression of the electroporated plasmids. Consequently, neurons revealed by

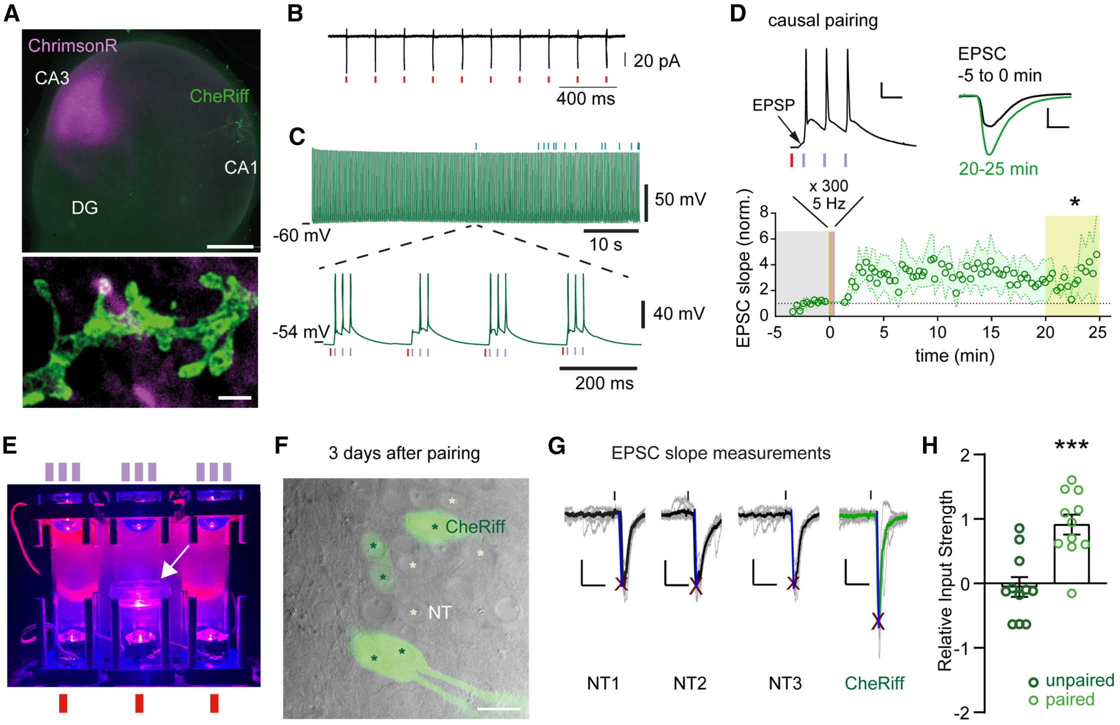

Figure 3. All-optical tLTP in hippocampal slice culture lasts for 3 days

(A) Transfection/transduction strategy for hippocampal STDP. Top: wide-field image of hippocampal slice culture shows cluster of virally transduced CA3 pyramidal cells (magenta) and a single transfected CA1 pyramidal cell (green). Bottom: STED image (z projection) of CheRiff-expressing dendrites (green) overlaid with confocal image of presynaptic ChrimsonR-expressing boutons (magenta), showing a putative synaptic contact (white). Scale bars: 500 μm and 1 μm.

(B) Representative cell-attached recordings of Flex-ST-ChrimsonR, co-expressed with SYP-HRP-Cre CA3 neurons, recorded in culture medium. CA3 Syp-HRP-Cre + Flex-ST-ChrimsonR neuron fires single action potential in response to red light pulses (red tics).

(C) Representative current-clamp recording of a CA1 neuron while applying paired optical stimulation. Blue ticks above the trace indicate stimulations where only 2 postsynaptic spikes were triggered instead of 3 (see expanded timescale below). In this example, 286 out of 300 paired stimulations were flawless.

(D) After oSTDP induction (left inset, scale bars: 20 mV, 20 ms), the slope of EPSCs increases (right inset, scale bars: 200 pA, 10 ms). Red tic indicates the light pulse (7 ms conduction delay from CA3 to CA1). Paired t test, $p = 0.044$, $n = 4$ slice cultures. Data are plotted as mean ± SEM.

(E) Three LED towers for in-incubator stimulation. The middle tower contains a 35 mm Petri dish with cell culture insert (white arrow). Ticks symbolize the three 405 nm light pulses from the top LED and the single 635 nm pulse from the bottom LED.

(F) Dodt contrast and epifluorescence image of CA1. CheRiff neurons (green asterisks) were distinguished from non-transfected (NT) neighbors (white asterisks) by their somatic fluorescence (green). Scale bar: 36 μm.

(G) Calculation of input strength. Vertical lines indicate presynaptic optical stimulation (1 ms, 594 nm, identical light intensity was used to stimulate sequentially patched NT and CheRiff cells in each slice). For each neuron, the 20%–60% slope was measured on the averaged EPSC (thick traces) from 10 consecutive stimulations (gray traces). Red crosses show auto-detected peaks. Scale bars: 25 ms, 50 pA.

(H) Effect of paired optical stimulation on input strength. Each circle shows the EPSC slope of a CheRiff-expressing neuron normalized to the EPSC slope in 2–3 NT neighbors. The tLTP group (light green) was assessed 3 days after causal pairing, and the control group (dark green) received no paired stimulation. Unpaired t test, $p = 0.0002$, $n = 11$, 11 neurons. Bars show mean ± SEM.

DAB staining corresponded to mKate2 fluorescence several days before fixation (Figure 2D).

Step 2: Validation of all-optical tLTP induction

For oSTDP induction, we used two channelrhodopsins with largely separated activation spectra, ChrimsonR and CheRiff.[25,26] Previously, we carefully characterized their properties and found that low-intensity violet light pulses (~1 mW mm^{-2}, 2 ms) selectively generate single action potentials in CheRiff-expressing neurons, while high-intensity orange to red light pulses (~7 mW mm^{-2}, 2 ms) selectively spike ChrimsonR-expressing neurons[4] (Figure 1, step 1). Slice cultures with virally transduced CA3 neurons and electroporated CA1 neurons (Figure 3A) were stimulated with a 594 nm laser positioned through the condenser to illuminate CA3 or a red light-emitting diode (LED) coupled through the objective. CheRiff-expressing CA1 neurons were stimulated by a 405 nm LED coupled through the objective. Using the exact combinations of viral vectors and plasmids described in step 1, we verified that ChrimsonR/syp-HRP-expressing CA3 neurons faithfully followed red light flashes with single action potentials in cell-attached recordings (Figure 3B). When recording from CA1 neurons, a 2 ms, 594 nm light pulse elicited an EPSC of 100–200 pA. To induce tLTP, the 594 nm light pulse was paired with a burst of 3 violet light flashes, delayed so that the first spike in the CA1 neuron occurred 10 ms after the onset of the EPSC (+10 ms causal pairing). Repeated 300 times

at 5 Hz, this spike/burst stimulation pattern produced EPSCs and 3 spikes in CheRiff/dAPEX2-expressing CA1 neurons and induced tLTP (Figures 3C and 3D).

Step 3: Optogenetic stimulation in the incubator

To follow the stimulated synapses over several days or before fixing for ultrastructural analysis, we optically stimulated slice cultures inside the cell culture incubator (Figure 3E). As there is no baseline to compare EPSCs after STDP, the EPSC slope of paired CA1 neurons is normalized to the average EPSC slope of several (3–5) neighboring NT neurons (NT EPSCs; Figures 3F and 3G). The relative input strength is calculated as

$$\text{relative input strength} = \frac{\text{EPSC slope} - \text{average NT EPSC slope}}{½(\text{EPSC slope}+\text{average NT slope})}.$$

As previously reported,[4] the relative synaptic input strength to CheRiff-expressing CA1 neurons was stronger compared to nearby NT neurons 3 days after causal pairing (Figures 3G and 3H). Thus, in organotypic slices, tLTP persists for at least 3 days (late tLTP). Importantly, too many simultaneously spiking CA3 neurons will directly drive spiking in the postsynaptic CA1 neurons, even in the absence of CheRiff and violet light.[4] In this case, the NT neurons will also undergo STDP, which means that the specificity that only connections between the ChrimsonR-expressing presynaptic neurons and the CheRiff-expressing postsynaptic neurons are potentiated will be lost. LTP is still induced under these conditions, but it is not possible to use unlabeled postsynaptic neurons as a reference group. Thus, if the goal is to compare the ultrastructure of "paired" synapses with that of neighboring "control" synapses in CA1, it is a requirement that relatively few CA3 neurons express ChrimsonR/syp-HRP.

Steps 4–9: Preparation of tissue for TEM and serial sectioning

After plasticity induction, each slice culture was fixed and processed for EM examination. We tested several staining protocols for syp-HRP and dAPEX2. We found that metal-enhanced DAB staining followed by osmification was necessary to reliably visualize both the axonal boutons of CA3 neurons and the spines and dendrites of CA1 neurons. Briefly, after the DAB reaction using metal-enhanced DAB substrate (Thermo 34065), the section was trimmed under visual control using the stained CA1 neurons as a guide. The trimmed specimens were then fixed with 1% (w/v) osmium tetroxide, dehydrated in an ascending series of ethanol, and embedded in Epon 812. Several rounds of trimming were required to center the brown DAB-stained CA1 neurons in the blocks, minimizing the area to be searched for synaptic contacts. Semi-thin sections were cut and immediately inspected for DAB-stained somata (Figure 1, step 7). After further trimming of the edges of the mounted block, serial ultrathin sections were cut. For quality control, we sectioned separate blocks containing the somata of stained CA3 and CA1 neurons and inspected them for abnormalities (Figure S2). Organelles such as the Golgi apparatus, mitochondria, and rough ER were abundant in the cytoplasm, and nuclei appeared normal. Note that dAPEX2 creates a granular precipitate of electron-dense particles in the nuclei and cytoplasm of CA1 neurons that is not associated with any particular biological structure.

Step 10: Analysis of synaptic ultrastructure after STDP

Labeled synaptic vesicles in channelrhodopsin-expressing axons appeared as distinct, electron-dense dots. This allowed for the identification of stimulated axons and their boutons (Figure 4). The somata (Figure S2), dendrites, and spines of HRP-labeled postsynaptic neurons were filled with granular electron-dense material that was easy to recognize in ultrathin sections. Potential synaptic connections were identified as contacts between labeled presynaptic and postsynaptic compartments (Figures 4A and S3). Both single- and double-labeled synapses could be identified in the same specimen (Figure 4B), which provided important internal controls: contacts of labeled boutons with unlabeled spines experienced presynaptic activity and glutamate release, but not pairing with postsynaptic spikes. Labeled spines in contact with unlabeled boutons experienced triplets of back-propagating action potentials, but not simultaneous glutamate release. These forms of activity are not expected to induce strong long-term plasticity, though some degree of heterosynaptic plasticity is possible. As a second oSTDP group, we analyzed synapses from a slice culture that we stimulated in anti-causal sequence: three postsynaptic action potentials were followed by one presynaptic action potential (Figure S4). Anti-causal stimulation is known to induce early tLTD.[4]

A comparison of the size of presynaptic boutons revealed that tLTP synapses had a larger cross-sectional area than unlabeled synapses (Figure 4E), resulting in longer axon-spine interfaces (ASIs). Interestingly, the ASI became strongly curved after tLTP, suggesting that the activated spine heads began wrapping around the active presynaptic boutons (Figure 4E). Actin polymerization inside spine heads is indeed triggered by NMDAR-mediated calcium influx,[27] and enlarged, cup-shaped spines have frequently been reported after LTP protocols.[28,29] Not all bouton-spine contacts had a clearly identifiable postsynaptic density (PSD) in the inspected sections (Figures S3 and S4). However, the median length of double-labeled PSDs after tLTP increased significantly compared to PSDs in contact with non-activated boutons (post only; Figure 4E). The PSDs of tLTP synapses were also much larger than the PSDs of the tLTD group, reflecting the well-established functional consequences of causal versus anti-causal stimulation.[1,4] Due to our limited 2D dataset, we could not analyze spine morphology in more detail. Since LTP is believed to affect the geometry of the spine neck,[16] generating 3D data of synapses after oSTDP is an exciting prospect.

In summary, the task of finding the connecting synapses between a small number of presynaptic neurons and a specific CA1 neuron is difficult but not impossible. Based on the excitatory postsynaptic potential (EPSP) amplitude in response to presynaptic optogenetic stimulation, a typical miniature EPSC (mEPSC) amplitude of 20 pA,[30] and an average release probability of 0.5, we estimate ∼20 light-stimulated synapses onto a single postsynaptic neuron, corresponding to about 0.1%–0.2% of spines.[31] Thus, we needed to inspect approximately 1,000

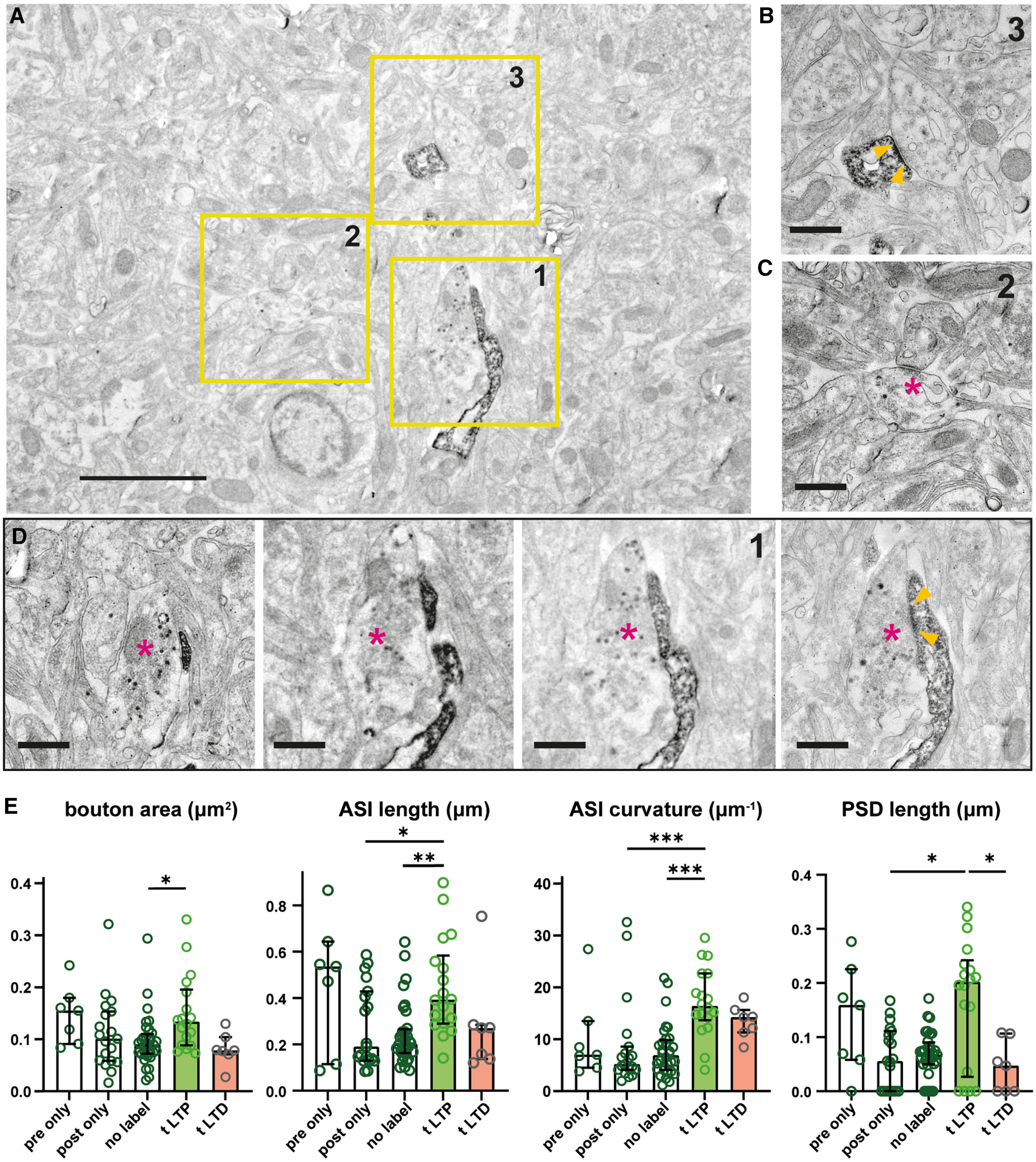

Figure 4. Causal pairing drives expansion of spine-bouton interface and postsynaptic density

(A) In the same field of view (FOV), a double-labeled synapse (1) and single-labeled synapses (2 and 3) are visible.

(B) High-resolution image of the labeled spine (3) in synaptic contact with unlabeled bouton. Yellow arrow tips delimit postsynaptic density (PSD).

(C) High-resolution image of bouton (2, magenta asterisk) with two labeled vesicles in contact with unlabeled postsynaptic element.

(D) Serial sections through the double-labeled synapse (red asterisk: bouton, blue arrow: spine). The PSD is visible in the last section (yellow arrow heads). Scale bars: 2 μm, 500 nm.

(E) Analysis of bouton area, axon-spine interface (ASI) length, ASI curvature, and PSD length (pre only: $n = 7$; post only: $n = 19$; no label: $n = 32$; tLTP: $n = 18$; tLTD: $n = 7$ synapses). Bars show median ± quartiles. ANOVA followed by Kruskal Wallis tests (*$p < 0.05$, **$p < 0.01$, and ***$p < 0.001$).

See also Figures S3 and S4.

spines from a single CA1 neuron to find one double-labeled synapse. In practice, we increased our odds by targeted search on oblique dendrites in *stratum radiatum*, where CA3 axons form most of their contacts.

DISCUSSION

We present a workflow to first induce tLTP with light pulses and subsequently localize and study potentiated Schaffer collateral

synapses in ultrathin serial sections. Compared to previous approaches to studying the ultrastructure of potentiated synapses, our method has several advantages: oSTDP is induced inside a cell culture incubator at 37°C, avoiding the insertion of electrodes and other perturbations between plasticity induction and fixation. The resulting tLTP lasts for days, providing a remarkably long time window for studying ultrastructural changes. Using targeted peroxidase labels, each labeled synaptic structure could be assigned unambiguously to presynaptic or postsynaptic neurons and the associated stimulation pattern. This made it easy to identify essential control groups of synapses (presynaptic stimulation only, postsynaptic stimulation only, or unstimulated) present within each tissue block. DAB staining and sample trimming under a bright-field microscope are basic procedures; correlative fluorescence imaging or antibody staining was not required for our approach. Thus, we could use a strong fixation protocol to obtain high-contrast images. All viral vectors are compatible with the lowest biosafety level (BSL-1). By combining optogenetic tools with peroxidase-based genetic tags using Cre/*LoxP*, we present a method to study the ultrastructural changes of tLTP from minutes to days after plasticity induction. 10 min after the induction of tLTP, we found evidence for rapid growth of PSDs at double-labeled synapses and strongly curved ASIs that suggest cup-shaped spines. As a future perspective, it may be possible to induce oSTDP in a suitably equipped high-pressure freezing machine[32] to study even faster changes in synaptic ultrastructure.

Limitations of the study

The main limitation of our method is its low throughput, limited by the time required to manually search and locate double-labeled synapses with conventional serial-section TEM. Deep learning-based methods have proven extremely powerful for the automated tracing and detection of synapses in EM images[33,34] and could be applied to this problem. A further advance would be to combine our stimulation and labeling approach with volume EM methods such as array electron tomography,[35] serial block-face EM,[36] or focused ion beam milling.[37,38] Since DAB staining of tissue blocks is straightforward (no antibody staining required), our approach seems particularly well suited for these high-throughput methods. We did not, however, test this idea. A second limitation is the possibility of missing small presynaptic boutons of labeled CA3 neurons, as only about 20% of presynaptic vesicles become electron dense.[21] The comparatively large size of labeled boutons (pre only; Figure 4E) is probably a reflection of this bias. Tracking axons through serial sections or 3D volumes will mitigate this risk, as nearby boutons containing labeled vesicles will signal that an adjacent unlabeled bouton was also active during plasticity induction.

RESOURCE AVAILABILITY

Lead contact

Requests for resources, reagents, and further information should be directed to the lead contact, Thomas G. Oertner (thomas.oertner@zmnh.uni-hamburg.de).

Materials availability

The pAAV-Syn-SYP-HRP-IRES-Cre plasmid generated in this study has been deposited to Addgene (#231252). Recombinant AAVs used in this study are available from the lead contact upon request.

Data and code availability

- TEM images and electrophysiological data generated during this study will be shared by the lead contact upon request.
- Custom code for electrophysiology analysis has been deposited at Zenodo and is publicly available (https://doi.org/10.5281/zenodo.15976791).
- Any additional information required to reanalyze the data reported in this paper is available from the lead contact upon request.

ACKNOWLEDGMENTS

We thank Jan Schröder, Iris Ohmert, Emanuela Szpotowicz, and Saskia Siegel for their excellent technical assistance. Bas von Bommel performed STED microscopy at the UKE Microscopy Imaging Facility with Virgilio Failla. Ingke Braren from the UKE Vector Facility produced rAAVs. pAAV-hSyn-DIO-ChrimsonR-mRuby2-ST was a gift from Hillel Adesnik (Addgene plasmid #105448). pAAV-syn-SYP-HRP (Addgene plasmid #117185) and pAAV-EF1α-dAPEX2 (Addgene plasmid #117173) were gifts from David Ginty. CheRiff (Addgene #51697) was a gift from Adam Cohen, and ChrimsonR (Addgene #59171) was a gift from Edward Boyden. Funding was received from Deutsche Forschungsgemeinschaft grants SFB 936 (178316478) and FOR 2419 (278170285) and the European Research Council (ERC-Synergy 951515).

AUTHOR CONTRIBUTIONS

T.G.O., M.S., and C.E.G. conceived the study, obtained funding, directed the work, and wrote the first draft together with R.W. R.W. performed the experiments; tested plasmids and viruses; prepared tissue, including DAB staining and trimming for EM; analyzed data; and produced the figures. M.S. produced serial sections and performed EM, and M.A. established oSTDP. All authors edited and approved the final manuscript.

DECLARATION OF INTERESTS

The authors declare no competing interests.

STAR★METHODS

Detailed methods are provided in the online version of this paper and include the following:

- KEY RESOURCES TABLE
- EXPERIMENTAL MODEL AND STUDY PARTICIPANT DETAILS
  - Animals
- METHOD DETAILS
  - Organotypic slice culture
  - Transduction of neurons in rat hippocampal slice culture
  - Electrophysiology
  - Optogenetic induction of spike-timing-dependent plasticity
  - Immunohistochemistry
  - Confocal and stimulated emission depletion (STED) microscopy
  - Sample preparation for electron microscopy
  - Transmission electron microscopy and ultrastructural analysis
- QUANTIFICATION AND STATISTICAL ANALYSIS

SUPPLEMENTAL INFORMATION

Supplemental information can be found online at https://doi.org/10.1016/j.crmeth.2025.101142.

Received: February 28, 2025
Revised: June 17, 2025
Accepted: July 30, 2025
Published: August 25, 2025

REFERENCES

1. Bi, G.Q., and Poo, M.M. (1998). Synaptic Modifications in Cultured Hippocampal Neurons: Dependence on Spike Timing, Synaptic Strength, and Postsynaptic Cell Type. J. Neurosci. *18*, 10464–10472. https://doi.org/10.1523/JNEUROSCI.18-24-10464.1998.
2. Markram, H., Lübke, J., Frotscher, M., and Sakmann, B. (1997). Regulation of Synaptic Efficacy by Coincidence of Postsynaptic APs and EPSPs. Science *275*, 213–215. https://doi.org/10.1126/science.275.5297.213.
3. Debanne, D., Gähwiler, B.H., and Thompson, S.M. (1998). Long-term synaptic plasticity between pairs of individual CA3 pyramidal cells in rat hippocampal slice cultures. J. Physiol. *507*, 237–247. https://doi.org/10.1111/j.1469-7793.1998.237bu.x.
4. Anisimova, M., Van Bommel, B., Wang, R., Mikhaylova, M., Wiegert, J.S., Oertner, T.G., and Gee, C.E. (2022). Spike-timing-dependent plasticity rewards synchrony rather than causality. Cereb. Cortex *33*, 23–34. https://doi.org/10.1093/cercor/bhac050.
5. Motta, A., Berning, M., Boergens, K.M., Staffler, B., Beining, M., Loomba, S., Hennig, P., Wissler, H., and Helmstaedter, M. (2019). Dense connectomic reconstruction in layer 4 of the somatosensory cortex. Science *366*, eaay3134. https://doi.org/10.1126/science.aay3134.
6. Bartol, T.M., Jr., Bromer, C., Kinney, J., Chirillo, M.A., Bourne, J.N., Harris, K.M., and Sejnowski, T.J. (2015). Nanoconnectomic upper bound on the variability of synaptic plasticity. eLife *4*, e10778. https://doi.org/10.7554/eLife.10778.
7. Sorra, K.E., and Harris, K.M. (1993). Occurrence and three-dimensional structure of multiple synapses between individual radiatum axons and their target pyramidal cells in hippocampal area CA1. J. Neurosci. *13*, 3736–3748. https://doi.org/10.1523/JNEUROSCI.13-09-03736.1993.
8. Kwon, H.-B., and Sabatini, B.L. (2011). Glutamate induces de novo growth of functional spines in developing cortex. Nature *474*, 100–104. https://doi.org/10.1038/nature09986.
9. Richards, D.A., Mateos, J.M., Hugel, S., de Paola, V., Caroni, P., Gähwiler, B.H., and McKinney, R.A. (2005). Glutamate induces the rapid formation of spine head protrusions in hippocampal slice cultures. Proc. Natl. Acad. Sci. USA *102*, 6166–6171. https://doi.org/10.1073/pnas.0501881102.
10. Willig, K.I., Wegner, W., Müller, A., Calvet-Fournier, V., and Steffens, H. (2021). Multi-label in vivo STED microscopy by parallelized switching of reversibly switchable fluorescent proteins. Cell Rep. *35*, 109192. https://doi.org/10.1016/j.celrep.2021.109192.
11. Watanabe, S., Punge, A., Hollopeter, G., Willig, K.I., Hobson, R.J., Davis, M.W., Hell, S.W., and Jorgensen, E.M. (2011). Protein localization in electron micrographs using fluorescence nanoscopy. Nat. Methods *8*, 80–84. https://doi.org/10.1038/nmeth.1537.
12. Desmond, N.L., and Levy, W.B. (1983). Synaptic correlates of associative potentiation/depression: an ultrastructural study in the hippocampus. Brain Res. *265*, 21–30. https://doi.org/10.1016/0006-8993(83)91329-X.
13. Ostroff, L.E., Fiala, J.C., Allwardt, B., and Harris, K.M. (2002). Polyribosomes Redistribute from Dendritic Shafts into Spines with Enlarged Synapses during LTP in Developing Rat Hippocampal Slices. Neuron *35*, 535–545. https://doi.org/10.1016/S0896-6273(02)00785-7.
14. Bell, M.E., Bourne, J.N., Chirillo, M.A., Mendenhall, J.M., Kuwajima, M., and Harris, K.M. (2014). Dynamics of nascent and active zone ultrastructure as synapses enlarge during long-term potentiation in mature hippocampus. J. Comp. Neurol. *522*, 3861–3884. https://doi.org/10.1002/cne.23646.
15. Rey, S., Marra, V., Smith, C., and Staras, K. (2020). Nanoscale Remodeling of Functional Synaptic Vesicle Pools in Hebbian Plasticity. Cell Rep *30*, 2006–2017.e3. https://doi.org/10.1016/j.celrep.2020.01.051.
16. Tønnesen, J., Katona, G., Rózsa, B., and Nägerl, U.V. (2014). Spine neck plasticity regulates compartmentalization of synapses. Nat. Neurosci. *17*, 678–685. https://doi.org/10.1038/nn.3682.
17. Kuwajima, M., Ostrovskaya, O.I., Cao, G., Weisberg, S.A., Harris, K.M., and Zemelman, B.V. (2020). Ultrastructure of light-activated axons following optogenetic stimulation to produce late-phase long-term potentiation. PLoS One *15*, e0226797. https://doi.org/10.1371/journal.pone.0226797.
18. Ayuso-Jimeno, I.P., Ronchi, P., Wang, T., Gallori, C.E., and Gross, C.T. (2022). Identifying long-range synaptic inputs using genetically encoded labels and volume electron microscopy. Sci. Rep. *12*, 10213. https://doi.org/10.1038/s41598-022-14309-4.
19. Horstmann, H., Vasileva, M., and Kuner, T. (2013). Photooxidation-Guided Ultrastructural Identification and Analysis of Cells in Neuronal Tissue Labeled with Green Fluorescent Protein. PLoS One *8*, e64764. https://doi.org/10.1371/journal.pone.0064764.
20. Sonomura, T., Furuta, T., Nakatani, I., Yamamoto, Y., Unzai, T., Matsuda, W., Iwai, H., Yamanaka, A., Uemura, M., and Kaneko, T. (2013). Correlative analysis of immunoreactivity in confocal laser-scanning microscopy and scanning electron microscopy with focused ion beam milling. Front. Neural Circuits *7*, 26. https://doi.org/10.3389/fncir.2013.00026.
21. Zhang, Q., Lee, W.-C.A., Paul, D.L., and Ginty, D.D. (2019). Multiplexed peroxidase-based electron microscopy labeling enables simultaneous visualization of multiple cell types. Nat. Neurosci. *22*, 828–839. https://doi.org/10.1038/s41593-019-0358-7.
22. Sauer, B. (1987). Functional expression of the cre-lox site-specific recombination system in the yeast Saccharomyces cerevisiae. Mol. Cell Biol. *7*, 2087–2096.
23. Martell, J.D., Deerinck, T.J., Lam, S.S., Ellisman, M.H., and Ting, A.Y. (2017). Electron microscopy using the genetically encoded APEX2 tag in cultured mammalian cells. Nat. Protoc. *12*, 1792–1816. https://doi.org/10.1038/nprot.2017.065.
24. Wiegert, J.S., Gee, C.E., and Oertner, T.G. (2017). Single-Cell Electroporation of Neurons. Cold Spring Harb. Protoc. *2017*, pdb-prot094904. https://doi.org/10.1101/pdb.prot094904.
25. Hochbaum, D.R., Zhao, Y., Farhi, S.L., Klapoetke, N., Werley, C.A., Kapoor, V., Zou, P., Kralj, J.M., Maclaurin, D., Smedemark-Margulies, N., et al. (2014). All-optical electrophysiology in mammalian neurons using engineered microbial rhodopsins. Nat. Methods *11*, 825–833. https://doi.org/10.1038/nmeth.3000.
26. Klapoetke, N.C., Murata, Y., Kim, S.S., Pulver, S.R., Birdsey-Benson, A., Cho, Y.K., Morimoto, T.K., Chuong, A.S., Carpenter, E.J., Tian, Z., et al. (2014). Independent optical excitation of distinct neural populations. Nat. Methods *11*, 338–346. https://doi.org/10.1038/nmeth.2836.
27. Bosch, M., and Hayashi, Y. (2012). Structural plasticity of dendritic spines. Curr. Opin. Neurobiol. *22*, 383–388. https://doi.org/10.1016/j.conb.2011.09.002.
28. Kulik, Y.D., Watson, D.J., Cao, G., Kuwajima, M., and Harris, K.M. (2019). Structural plasticity of dendritic secretory compartments during LTP-induced synaptogenesis. eLife *8*, e46356. https://doi.org/10.7554/eLife.46356.
29. Sun, Y., Smirnov, M., Kamasawa, N., and Yasuda, R. (2021). Rapid Ultrastructural Changes in the PSD and Surrounding Membrane after Induction of Structural LTP in Single Dendritic Spines. J. Neurosci. *41*, 7003–7014. https://doi.org/10.1523/JNEUROSCI.1964-20.2021.
30. Baxter, A.W., and Wyllie, D.J.A. (2006). Phosphatidylinositol 3 kinase activation and AMPA receptor subunit trafficking underlie the potentiation of miniature EPSC amplitudes triggered by the activation of L-type calcium channels. J. Neurosci. *26*, 5456–5469. https://doi.org/10.1523/JNEUROSCI.4101-05.2006.
31. Megías, M., Emri, Z., Freund, T.F., and Gulyás, A.I. (2001). Total number and distribution of inhibitory and excitatory synapses on hippocampal

CA1 pyramidal cells. Neuroscience *102*, 527–540. https://doi.org/10.1016/s0306-4522(00)00496-6.

32. Chakrabarti, R., Jaime Tobón, L.M., Slitin, L., Redondo Canales, M., Hoch, G., Slashcheva, M., Fritsch, E., Bodensiek, K., Özçete, Ö.D., Gültas, M., et al. (2022). Optogenetics and electron tomography for structure-function analysis of cochlear ribbon synapses. eLife *11*, e79494. https://doi.org/10.7554/eLife.79494.

33. Schmidt, M., Motta, A., Sievers, M., and Helmstaedter, M. (2024). RoboEM: automated 3D flight tracing for synaptic-resolution connectomics. Nat. Methods *21*, 908–913. https://doi.org/10.1038/s41592-024-02226-5.

34. Su, F., Wei, M., Sun, M., Jiang, L., Dong, Z., Wang, J., and Zhang, C. (2023). Deep learning-based synapse counting and synaptic ultrastructure analysis of electron microscopy images. J. Neurosci. Methods *384*, 109750. https://doi.org/10.1016/j.jneumeth.2022.109750.

35. Micheva, K.D., and Smith, S.J. (2007). Array tomography: a new tool for imaging the molecular architecture and ultrastructure of neural circuits. Neuron *55*, 25–36. https://doi.org/10.1016/j.neuron.2007.06.014.

36. Denk, W., and Horstmann, H. (2004). Serial block-face scanning electron microscopy to reconstruct three-dimensional tissue nanostructure. PLoS Biol. *2*, e329. https://doi.org/10.1371/journal.pbio.0020329.

37. Young, R.J., and Moore, M.V. (2005). Dual-Beam (FIB-SEM) Systems. In Introduction to Focused Ion Beams: Instrumentation, Theory, Techniques and Practice, L.A. Giannuzzi and F.A. Stevie, eds. (Springer US), pp. 247–268. https://doi.org/10.1007/0-387-23313-X_12.

38. Knott, G., Marchman, H., Wall, D., and Lich, B. (2008). Serial section scanning electron microscopy of adult brain tissue using focused ion beam milling. J. Neurosci. *28*, 2959–2964. https://doi.org/10.1523/JNEUROSCI.3189-07.2008.

39. Suter, B.A., O'Connor, T., Iyer, V., Petreanu, L.T., Hooks, B.M., Kiritani, T., Shepherd, G.M.G., and Svoboda, K. (2010). Ephus: Multipurpose Data Acquisition Software for Neuroscience Experiments. Front. Neural Circuits *4*, 100.

40. Gee, C.E., Ohmert, I., Wiegert, J.S., and Oertner, T.G. (2017). Preparation of Slice Cultures from Rodent Hippocampus. Cold Spring Harb. Protoc. *2017*, pdb-prot094888. https://doi.org/10.1101/pdb.prot094888.

41. Wiegert, J.S., Gee, C.E., and Oertner, T.G. (2017). Viral Vector-Based Transduction of Slice Cultures. Cold Spring Harb. Protoc. *2017*, pdb.prot094896. https://doi.org/10.1101/pdb.prot094896.

42. Mary, H., and Brouhard, G.J. (2019). Kappa (κ): Analysis of Curvature in Biological Image Data using B-splines. Preprint at BioRxiv, 852772. https://doi.org/10.1101/852772.

CellPress
OPEN ACCESS

STAR★METHODS

KEY RESOURCES TABLE

REAGENT or RESOURCE	SOURCE	IDENTIFIER
**Antibodies**		
ATTO 647N conjugated (camelid) anti-GFP	NanoTag	Cat# N0304-At647N-S, RRID: AB_2905516
Abberior STAR 580 (camelid) anti-RFP	NanoTag	Cat# N0401-Ab580-S, RRID: AB_2744601
Anti-Cre (mouse)	Millipore	Cat# MAB3120, RRID: AB_2085748
Anti-mouse Alexa Fluor 647 (goat)	Invitrogen	Cat# A-21236, RRID: AB_2535805
**Bacterial and virus strains**		
$AAV^{2/9}$ Syn-SYP-HRP-IRES-Cre	UKE Vector Core Fac.	N/A
$AAV^{2/9}$ hSyn-DIO-ChrimsonR-mRuby2-ST	UKE Vector Core Fac.	N/A
$AAV^{2/Rh10}$ Syn-ChrimsonR-tdT	UKE Vector Core Fac.	N/A
**Chemicals, peptides, and recombinant proteins**		
Triton X-100	Sigma-Aldrich	X100
PFA	Laborimpex	E15710
GA	Laborimpex	E16320
Osmium Tetroxide	Laborimpex	E19110
Sodium Cacodylate	Scientific Laboratory Supplies	C4945
Goat serum	Capricorn Scientific	GOA-1A
Glycidyl ether	Carl Roth	8619.2
MNA	Carl Roth	8639.2
DBA	Carl Roth	8623.2
DMP-30	Carl Roth	8621.1
**Critical commercial assays**		
Metal Reinforced DAB Substrate Kit	Thermo Fisher	Cat# 34065
**Experimental models: Organisms/strains**		
Wild-type Wistar (HsdCpb:Wu,Envigo) or Sprague-Dawley (Hsd:Sprague Dawley®SD® Inotiv) rats	Envigo	HsdCpb: Wu
**Recombinant DNA**		
pAAV-hSyn-CheRiff-eGFP	Addgene	Cat# 51697
pAAV-Syn-ChrimsonR-tdT	Addgene	Cat# 59171
pCI-Syn-mKate2-N	This study	Modified from Evrogen plasmid Cat# FP182
pAAV-Syn-SYP-HRP-IRES-Cre	This study	Addgene Cat #231252
pAAV-hSyn-DIO-ChrimsonR-mRuby2-ST	Addgene	Cat#105448
pAAV-EF1α- dAPEX2	Addgene	Cat#117173
**Software and algorithms**		
MATLAB	MathWorks	https://www.mathworks.com
Ephus	Suter et al.[39]	http://www.ephus.org
GraphPad Prism 10	Dotmatics	https://www.graphpad.com
Adobe Illustrator	Adobe	https://www.adobe.com
Imaris	OXFORD Instr.	https://imaris.oxinst.com
Fiji ImageJ	NIH	https://fiji.sc
Electrophysiology analysis tools	This paper	https://doi.org/10.5281/zenodo.15976791
Kappa	Gary J. Brouhard lab	https://github.com/fiji/Kappa

EXPERIMENTAL MODEL AND STUDY PARTICIPANT DETAILS

Animals

Wild-type Wistar or Sprague-Dawley rats were housed and bred at the University Medical Center Hamburg-Eppendorf (UKE) animal facility. Neonatal pups of either sex were sacrificed according to German Law (Tierschutzgesetz der Bundesrepublik Deutschland, TierSchG), with approval from the Behörde für Justiz und Verbraucherschutz (BJV)-Lebensmittelsicherheit und Veterinärwesen Hamburg and the animal care committee of the UKE.

METHOD DETAILS

Organotypic slice culture

Hippocampus slice cultures were prepared and kept in an antibiotic-free medium as previously described.[40] Rat pups at age P4 to P6 were decapitated under 80% CO_2/20% O_2 anesthesia. Hippocampi were dissected, leaving a portion of the entorhinal cortex attached, in ice-cold sterile slice culture dissection medium (in mM: 248 sucrose, 26 $NaHCO_3$, 10 glucose, 4 KCl, 5 $MgCl_2$, 2 kynurenic acid, and 0.001% phenol red. pH~7.4, osmolality 310 to 320 mOsm/kg, bubbled with sterile filtered 95% O_2/5% CO_2). 400 µm thick slices were cut from the dissected tissue with a McIllwain tissue chopper. Slices were cultured on 30 mm diameter porous membranes (Millipore PICMORG50), supplied with 1 mL of culture medium (for 500 mL: 394 mL Minimal Essential Medium (MEM, Sigma M7278), 100 mL heat-inactivated donor horse serum (Sigma H1138), 1 mM L-glutamine (Gibco 25030-024), 0.01 mg/mL insulin (Sigma I6634), 1.45 mL, 109 mM NaCl (Sigma S5150), 2 mM $MgSO_4$ (Fluka 63126), 1.44 mM $CaCl_2$ (Fluka 21114), 6 µM ascorbic acid (Fluka 11140), and 13 mM D-glucose (Fluka 49152), sterile filtered) in a cell culture incubator (37°C, 5% CO_2).

Transduction of neurons in rat hippocampal slice culture

Transfection was performed at 10–14 days *in vitro* (DIV) by CA1 single cell electroporation (pAAV-hsyn-CheRiff-eGFP 0.5 ng/µL, pCI-*syn*-mKate2 10 ng/µL, and dAPEX2 10 ng/µL) and transduction by CA3 local AAV injection ($AAV^{2/9}$-hSyn-DIO-ChrimsonR-mRuby2-ST, 1.05 × 10^{14} vg/mL and $AAV^{2/9}$-SynSYP-HRP-IRES-Cre, 6× 10^{13}vg/mL, measured by the WPRE sequence) according to published procedures.[24,41] In Figures 3G and 3H, CA3 neurons were transduced with $AAV^{2/Rh10}$- syn-ChrimsonR-tdTomato 4.38 × 10^{13} vg/mL and CA1 with hsyn-CheRiff-eGFP 0.5 ng/µL, pCI-*syn*-mKate2 10 ng/µL. Slice culture medium was partially changed (~2/3) every 2 to 3 days under dim yellow light (Osram LUMILUX CHIP control T8) to avoid unwanted activation of either the CheRiff or ChrimsonR. A mixture of $AAV^{2/Rh10}$ Syn-ChrimsonR-tdT 8.1 × 10^{12} vg/mL and $AAV^{2/Rh10}$ CMV-GFP 8.1 × 10^{12} vg/mL was used for testing the co-expression rate of two AAV-packaged constructs. Constructs were expressed for 4 days. Imaris 10.1.0 was used to analyze the co-expression rate of the two constructs (Figures 2A and 2B). Cre and DAPI channels were turned on and about 25 nuclei with both cre and DAPI staining were randomly selected from each image. Then the mRuby channel was turned on and the percentage of Cre+ mRuby-spots was determined. Next, the selection was started from mRuby and DAPI channels, and the percentage of mRuby+ Cre-spots was determined.

Electrophysiology

The electrophysiology setup was based on an Olympus BX61W1 epifluorescence microscope, Axopatch 200B amplifier (Axon) and a multi-wavelength fiber-coupled light source (Mightex, WFC). We used a 40x/1.0 Plan-Apochromat objective (Zeiss) which illuminates a field of 557 µm diameter. Optical stimulation experiments were performed 7–10 days after transduction. Whole-cell patch clamp recordings with oSTDP induction were performed in serum-free medium (in mM: 99% MEM (Sigma; M7278), 13 D-glucose, 109 NaCl, 2 $MgSO_4$, 1.44 $CaCl_2$, 1 L-glutamine (Gibco 25030), 0.006 ascorbic acid (Fluka 11140), 0.01 mg/mL insulin (Sigma I6634). pH 7.28, 310–318 mOsm/kg) to mimic in-incubator conditions. The intracellular solution for patch-clamping and electroporation contained (in mW): 135 K-gluconate, 4 $MgCl_2$, 4 Na_2-ATP, 0.4 Na-GTP, 10 Na_2-phosphocreatine, 3 ascorbate, and 10 HEPES, pH~7. 2 (solution was sterile filtered, aliquoted and stored at −20°C). CA1 pyramidal cells were voltage-clamped at −70 mV (liquid junction potential corrected), EPSCs were evoked by 594 nm laser pulses (2 ms, 0.05 Hz) directed through the condenser at CA3. After a short baseline period (<5 min after break-in), oSTDP was induced. During plasticity induction (one 594 nm 2 ms light pulse through the condenser (8 mW/mm^2) paired with three 405 nm 2 ms light pulses through the objective (1.2 mW/mm^2, 50 Hz), repeated 300 times at 5 Hz), the amplifier was switched to current clamp mode to allow spiking of the postsynaptic neuron. Next, the recording was switched back to voltage-clamp and further EPSCs were recorded (2 ms light pulses at 594 nm, 0.05 Hz) for 30 min. Series resistance and holding current were constantly monitored for quality control. ChrimsonR-induced spiking was assessed in cell-attached mode. EPSC amplitudes, slopes, and number of spikes were quantified using custom MATLAB scripts (R2016b).

Optogenetic induction of spike-timing-dependent plasticity

In-incubator oSTDP was induced according to published precedures.[4] Briefly, 1–2 days before stimulation, the last medium changed was performed and the slice culture was centered in a 35 mm culture dish. A stimulation tower with two LEDs was placed in the incubator. Two-color optical stimulation (identical to oSTDP induction during whole-cell recording) was controlled by a Master-8 stimulator (AMPI). Three days after in-incubator oSTDP, whole-cell recordings were performed in ACSF (in mM: 119 NaCl, 11 D-glucose, 2.5 KCl, 1 NaH_2PO_4, 4 $MgCl_2$, 4 $CaCl_2$, pH = 7.4, 305–315 mOsm/kg) aerated with Carbogen (95% O_2, 5% CO_2) at 31°C. CA1

pyramidal cells were voltage-clamped at −70 mV (liquid junction potential corrected) to record EPSCs triggered by 2 ms light pulses (594 nm) at 0.05 Hz.

Immunohistochemistry

Slice cultures were fixed in 4% PFA for 30 min, washed 3 times for 10 min in PBS and incubated for 1 h in blocking medium (PBS supplemented with 10% donor goat serum, 0.3% Triton X-100, 0.2% BSA). For images in Figure 2, anti-Cre mouse primary antibodies (Millipore MAB3120) were diluted 1:500, and anti-mouse Alexa Fluor 647 (goat) secondary antibodies (Invitrogen #A21236) were diluted 1:1000. After fixation and PBS washes, slices were incubated with primary antibody mix overnight at 4°C. The following day after 3 washes in PBS, slices were incubated with secondary antibody mix for 2 h, followed by washing, DAPI staining and mounting. For images in Figure 3A (bottom), anti-RFP (for ChrimsonR-tdTomato) and anti-GFP (for CheRiff-eGFP) conjugated camelid nanobodies (NanoTag) were diluted 1:250 in carrier solution (phosphate buffered saline (PBS) supplemented with 1% donor goat serum, 0.3% Triton X-100, 0.2% bovine serum albumin (BSA) to label boutons and spines (Figure 2A). After overnight incubation with the antibodies at 4°C, slices were washed 2 times in PBS, stained with DAPI (1:10000) and mounted in Mowiol, prepared according to manufacturer's protocol with anti-fading reagent DABCO.

Confocal and stimulated emission depletion (STED) microscopy

Gated-2D-STED images (Figure 2A) were acquired using an Abberior STED Expert line microscope with a 60x/1.4 P-Apo objective (Nikon). Fluorophores were excited with 561 nm and 640 nm laser lines. Fluorescence was collected by avalanche photodetectors through emission filters 615/20 (red channel) and 685/70 (far-red channel). Temporal gating was obtained with a Becker and Hickl SPC150 TCSPC board (8 ns window). Both channels (red, far-red) were acquired in confocal mode. In addition, the far-red channel was resolved with 2D-STED by use of a 775 nm depletion laser. Acquisitions were obtained sequentially with a 20 nm × 20 nm pixel size. Confocal images were the result of 6 accumulations, STED images of 18 accumulations acquired in line-mode. Confocal and STED images were merged in Fiji/ImageJ, the lookup table was linearly adjusted for each channel.

Sample preparation for electron microscopy

Each slice culture was fixed with 2.5% GA + 2% PFA at 37°C for 1 min, transferred to ice-cold 2.5% GA + 2% PFA for 1 h followed by 3 × 10 min cacodylate buffer wash (all wash steps below were performed with cacodylate buffer on ice. Slices can be kept in cacodylate buffer at 4°C for up to 3 weeks before DAB staining). Next, the slice was incubated with 20 mM glycine for 15 min, followed by 3 × 10 min wash. Endogenous peroxidase was blocked by 0.3% H_2O_2 incubation for 15 min, followed by 3 × 10 min wash. A metal-enhanced DAB substrate kit (Thermo Scientific) was used for DAB staining. Slice cultures were incubated with 1 x DAB reagent for 1 h to ensure full penetration before stabilized hydrogen peroxide solution (1:100) was added to initialize the DAB reaction. After 30–120 min of staining (depending on the expression level), slice cultures were 3 times washed and incubated with 3% GA overnight. The following day, the slice was washed again 3 times. Stained sections were micro-dissected to the CA1 or CA3 region and kept in cacodylate buffer at 4°C for up to 3 weeks. OsO_4 staining was then performed. The sections were washed in cacodylate buffer 2 × 5 min, followed by 20 min of 1% OsO_4 incubation on ice. Then, the sections were transferred to cacodylate buffer for washing for about an hour, with buffer replacement every 5 min.

To prepare for resin embedding, dissected specimens were dehydrated in an ethanol series (30%, 50%, 70%, 80%, 90%, 100% x 2, 15 min each), then moved to 100% propylene oxide (2 times, 10 min each). Next, specimens were pre-embedded in a 1:1 mixture of EPON 812 resin (Glycidyl ether hardener MNA 16.1 g, Glycidyl ether hardener DBA 8.025 g, Glycidyl ether accelerator DMP-30 0.5 g and Glycidyl ether 100 25.85 g) and Propylene oxide for 90 min, then transferred to a 2:1 mixture of EPON 812 resin and Propylene oxide for 2 h, then transferred to pure EPON resin and incubated overnight. The following day, embedded specimens were placed on a resin block and cured in an oven at 58°C for at least 48 h.

DAB staining results (Figure 1, step 5 and 6) were imaged on a Zeiss Axiophot with 10x/0.25 Achroplan objective and DFK37AUX250 camera (The Imaging Source), the micro-dissection was based on the DAB signal. After OsO_4 staining and embedding, semi-thin sections (~500 nm) were collected for further trimming (Figure 2, step 7): only the region containing the expected structure was kept for the collection of serial sections (e.g., *stratum radiatum* below a labeled CA1 soma, Figure 4).

A diamond knife was used for manual serial sectioning. Ultra-thin sections (50 nm) were collected on copper slot grids (0.4 × 2 mm). For achieving higher contrast, post-staining contrasting was performed. Each grid was placed on a drop of 100 μL uranyl acetate solution for 15 min, then washed in water, air dried in a grid box and moved to a drop of lead citrate for 1 min. After another washing and drying step, samples were ready for imaging. All contrasting steps were performed in a closed Petri dish.

Transmission electron microscopy and ultrastructural analysis

TEM images were acquired on a JEM-2100Plus electron microscope (JEOL) at 200 kV acceleration voltage with a JEOL recorder and CCD camera system (XAROSA, EMSIS), or on a TEM 900 electron microscope (Zeiss) at 80 kV acceleration voltage with a 4K digital camera (Tröndle, Moorenweis, Germany). Magnification was verified in different samples from 2,000× to 20,000 x. Panoramic overview images were obtained by tiling 64 TEM images obtained at lower magnification (7,000×), with 6 panoramas covering almost an entire ultrathin section (90,000 μm^2). Tiles containing labeled dendrites were identified by eye and high-resolution images (20,000 x) of those regions were acquired. Synapses were identified by searching for postsynaptic densities opposed by active zones and were

manually classified as unlabeled, pre-only, post-only, or double-labeled. Labeled presynaptic vesicles were distinguished from naturally occurring dense-core vesicles by their smaller size and characteristic gray value. If only a single labeled vesicle was identified in a bouton section, we checked consecutive sections or neighboring boutons of the same axon for additional labeled vesicles for confirmation. Identified synapses were morphometrically analyzed with ImageJ, the curvature of the axon-spine interface (ASI) was measured using the Kappa plug-in.[42] The center of curvature, defined as the inverse of the radius of the osculating circle (μm^{-1}), was on the presynaptic side in all cases.

QUANTIFICATION AND STATISTICAL ANALYSIS

Statistical tests and sample sizes are provided in the figure legends. Results with $p < 0.05$ were considered statistically significant. Statistical tests were performed and data were plotted using GraphPad Prism 10.

Cell Reports Methods, Volume *5*

Supplemental information

Ultrastructural analysis

# of synapses after induction

# of spike-timing-dependent plasticity

Rui Wang, Michaela Schweizer, Margarita Anisimova, Christine E. Gee, and Thomas G. Oertner

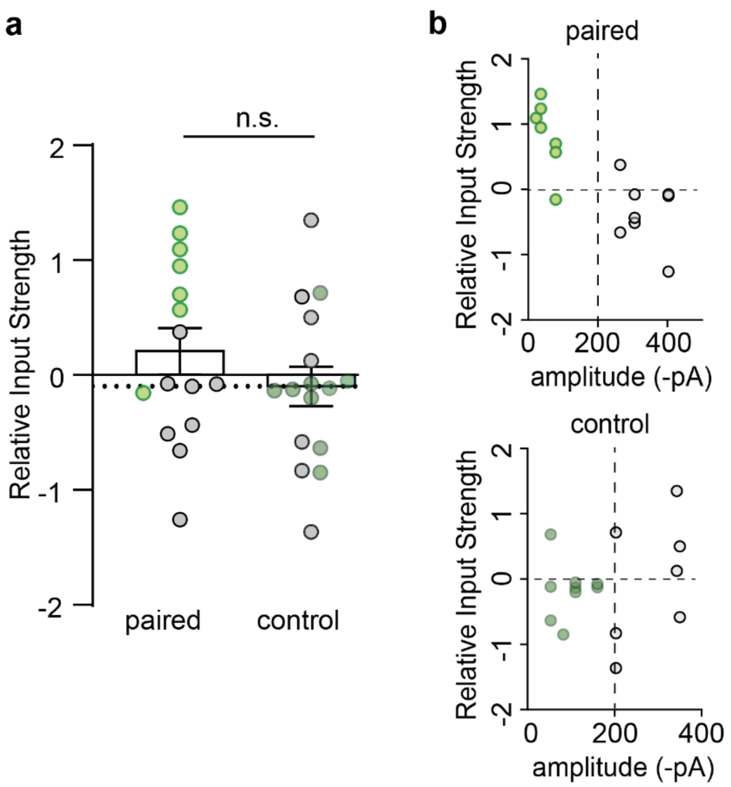

Figure S1. Activating a large number of presynaptic neurons prevents selective potentiation of paired synapses, related to Figure 3.

(**a**) Full dataset. Each dot indicates the EPSC amplitude of a CheRiff-expressing CA1 pyramidal cell relative to 2-3 non-transfected neighbors after causal pairing or without optical stimulation (control). No difference in input strength between the paired and the unpaired group relative to non-transfected neighbors. Green dots: average EPSC amplitude < 200 pA; black dots: average EPSC amplitude > 200 pA. n = 15, 16. Unpaired t-test, ns: not significant.
(**b**) Replot of data in (a) to show the relationship of input strength versus the averaged NT amplitude in paired (upper) and unpaired (lower) groups.
(**c**) All experiments with EPSC amplitude < 200 pA, indicating few ChrimsonR-expressing CA3 neurons. Significant input strengthening relative to non-transfected neighbors in the paired group only. n = 7, 9 neurons. Unpaired t-test, ***p < 0.001. Data plotted as mean ± SEM.

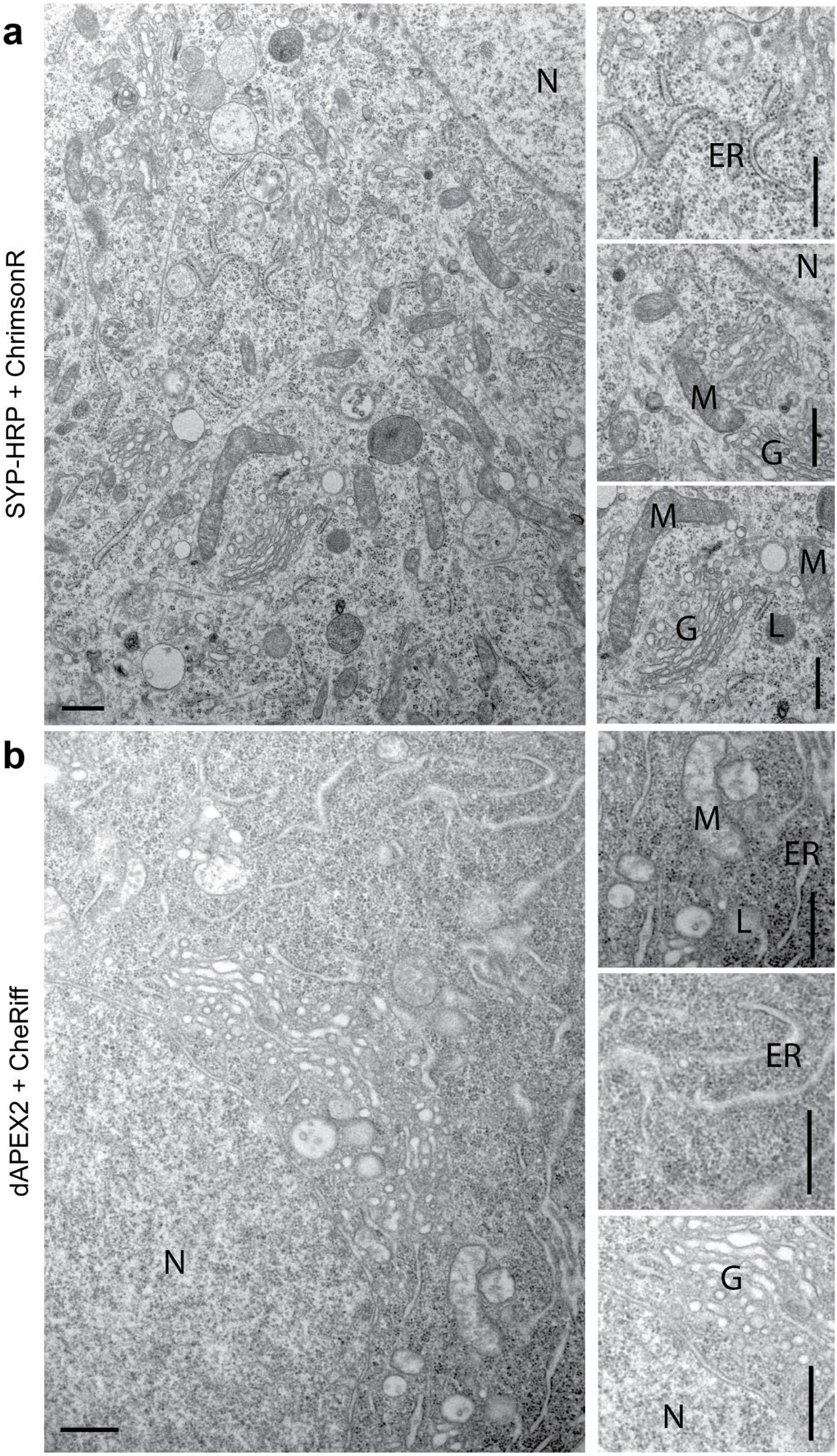
a
SYP-HRP + ChrimsonR
N
ER
N
M
G
M
M
G
L
b
dAPEX2 + CheRiff
N
M
ER
L
ER
G
N

Figure S2. Somatic ultrastructure of double-transduced neurons, related to Figure 4.

(**a**) CA3 pyramidal cell soma expressing SYP-HRP and ChrimsonR. Cytoplasm at the cell body including part of the nucleus (N). Due to synaptic vesicle targeting of HRP, no cytoplasmic DAB staining is visible in this neuron (see Fig. 4). Organelle morphology is well preserved (G: Golgi apparatus, M: mitochondria, L: lysosomes, ER: endoplasmic reticulum, N: nucleus). Scale bars: 500 nm.

(**b**) CA1 pyramidal cell soma expressing dAPEX2 and CheRiff. Membrane contrast is relatively low due to dAPEX2 condensation in the cytoplasm (dark granules). Organelle morphology is well preserved (G: Golgi apparatus, M: mitochondria, L: lysosomes, ER: endoplasmic reticulum, N: nucleus). Scale bars: 500 nm.

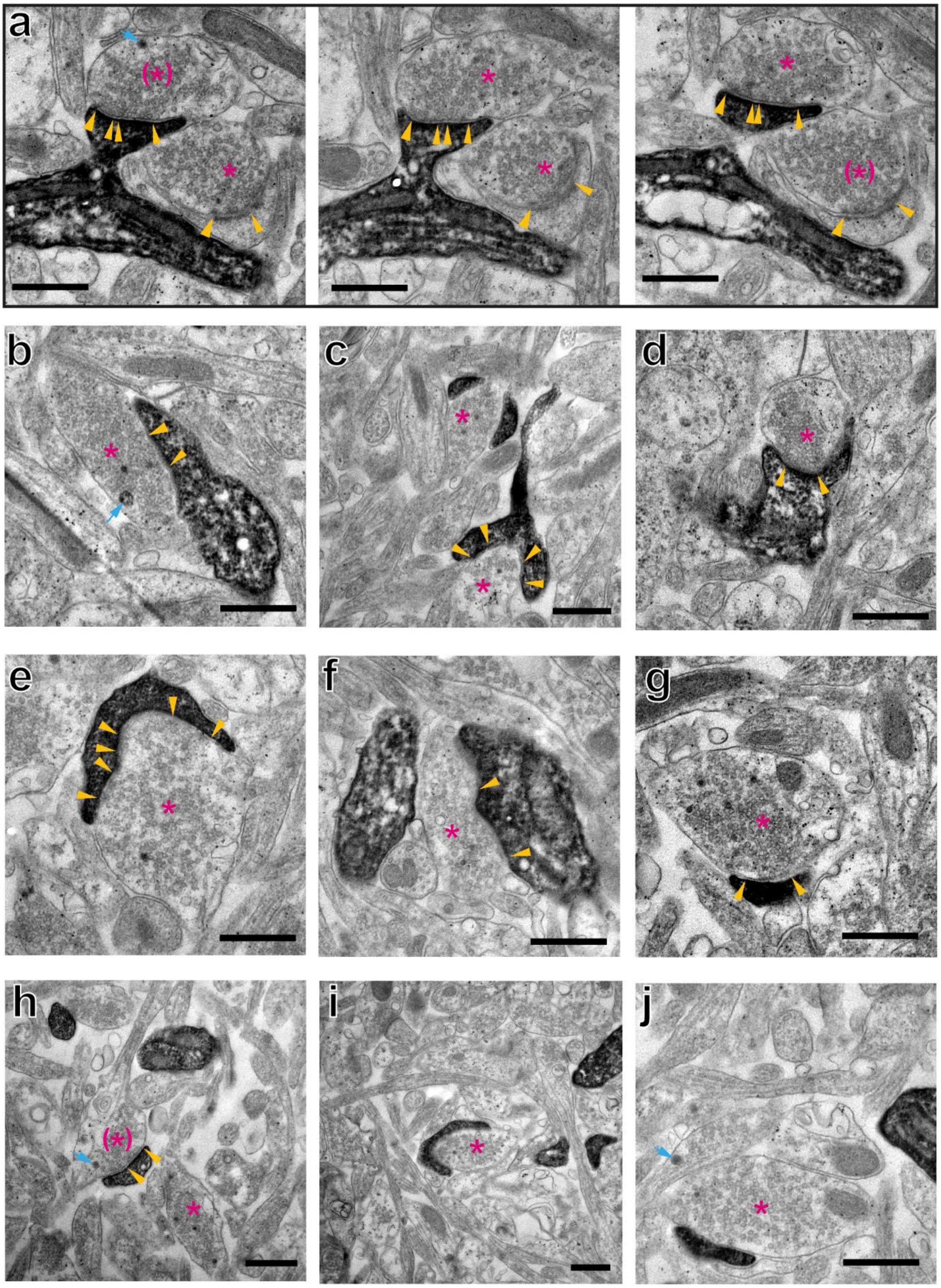

Figure S3. Double-labeled contacts after causal stimulation, related to Figure 4.

(**a**) Two spine synapses identified in 3 consecutive sections. Yellow arrowheads mark edge of PSDs. Magenta asterisks: Stimulated presynaptic terminal containing one or more labeled vesicles. Magenta asterisks in brackets: Stimulated terminal identified by labeled vesicles in other sections. Cyan arrow: dense core vesicle, not indicative of labeling. (**b**) – (**h**) Further examples of double-labeled synapses, identified in single sections. (**i**), (**j**) Putative synapses, PSD not identified. All scale bars: 250 nm.

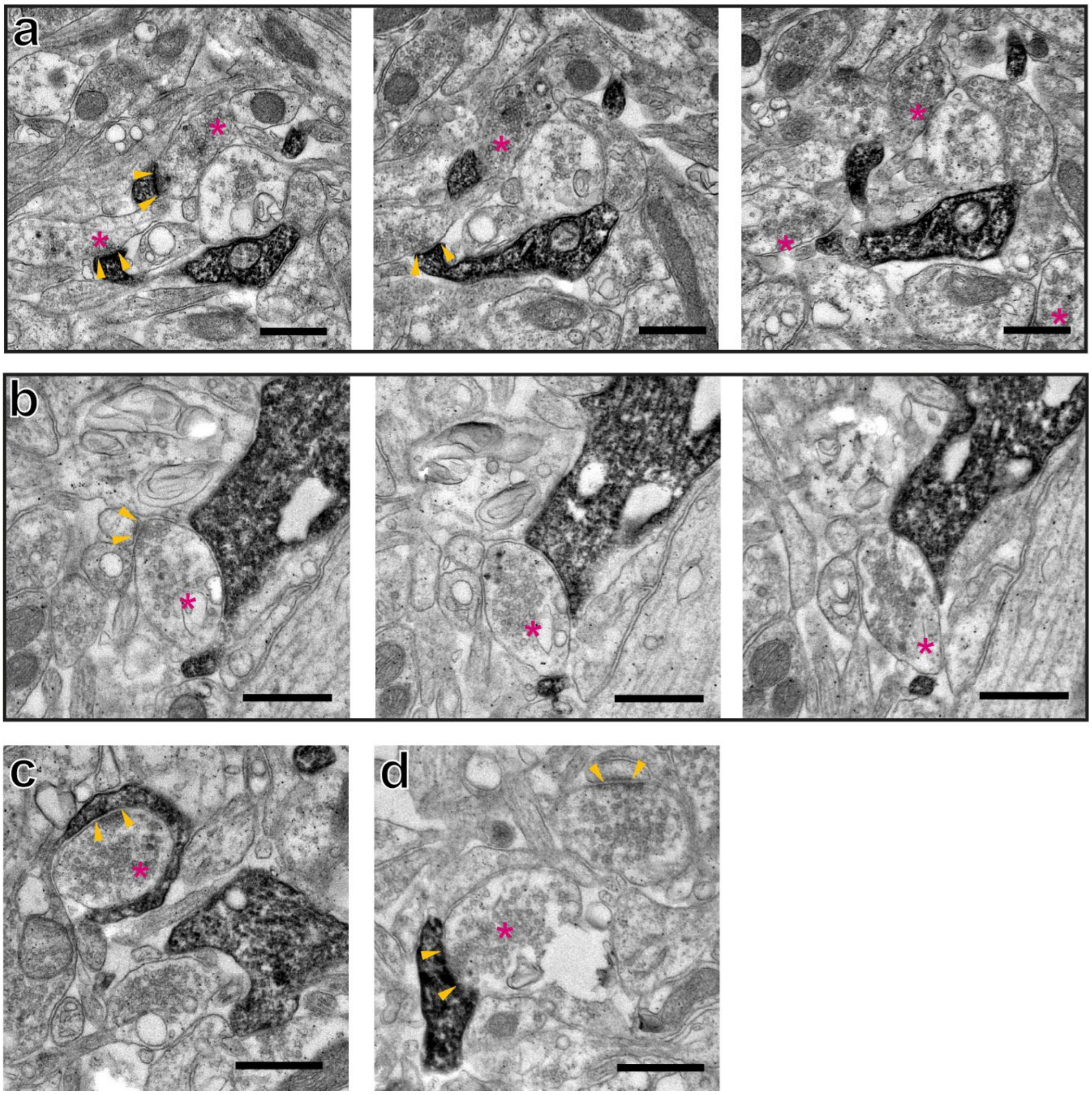

Figure S4. Double-labeled contacts after anti-causal stimulation, related to Figure 4.

(**a**) Two double-labeled spine synapses identified in 3 consecutive sections. Magenta asterisks: Stimulated presynaptic terminal containing one or more labeled vesicles. Yellow arrowheads denote PSD. (**b**) Pre-only synapse (Yellow arrowheads) with contact to a labeled postsynaptic dendrite, identified in 3 consecutive sections. (**c**),(**d**) Further examples of double-labeled synapses. All scale bars: 250 nm.
